# The microbiome and gene expression of honey bee workers are affected by a diet containing pollen substitutes

**DOI:** 10.1371/journal.pone.0286070

**Published:** 2023-05-19

**Authors:** J. Elijah Powell, Pierre Lau, Juliana Rangel, Ryan Arnott, Tyler De Jong, Nancy A. Moran

**Affiliations:** 1 Department of Integrative Biology, University of Texas at Austin, Austin, TX, United States of America; 2 Department of Entomology, Texas A&M University, College Station, TX, United States of America; 3 USDA-ARS, Pollinator Health in Southern Crop Ecosystem Research Unit, Stoneville, MS, United States of America; Institute of Apicultural Research, CHINA

## Abstract

Pollen is the primary source of dietary protein for honey bees. It also includes complex polysaccharides in its outer coat, which are largely indigestible by bees but can be metabolized by bacterial species within the gut microbiota. During periods of reduced availability of floral pollen, supplemental protein sources are frequently provided to managed honey bee colonies. The crude proteins in these supplemental feeds are typically byproducts from food manufacturing processes and are rarely derived from pollen. Our experiments on the impact of different diets showed that a simplified pollen-free diet formulated to resemble the macronutrient profile of a monofloral pollen source resulted in larger microbial communities with reduced diversity, reduced evenness, and reduced levels of potentially beneficial hive-associated bacteria. Furthermore, the pollen-free diet sharply reduced the expression of genes central to honey bee development. In subsequent experiments, we showed that these shifts in gene expression may be linked to colonization by the gut microbiome. Lastly, we demonstrated that for bees inoculated with a defined gut microbiota, those raised on an artificial diet were less able to suppress infection from a bacterial pathogen than those that were fed natural pollen. Our findings demonstrate that a pollen-free diet significantly impacts the gut microbiota and gene expression of honey bees, indicating the importance of natural pollen as a primary protein source.

## Introduction

Honey bee (*Apis mellifera*) nutrition is based on the intake of two resources that foragers collect from flowering plants: pollen, which provides proteins, lipids, and other nutrients (*i*.*e*., vitamins, and minerals) linked to physiological processes, and nectar, which provides the main source of energy in the form of simple carbohydrates [1, 2]. Each grain is made of a core cytoplasm comprised mostly of proteins, carbohydrates, and vitamins, surrounded by a durable coat called the pollenkitt [3]. This coat is made up of an outer exine layer composed of the inert lipid and phenolic biopolymer sporopollenin and an intine layer composed mostly of cellulose, pectin, and other polysaccharides [4].

Honey bee colonies are frequently managed in settings where pollen sources are inadequate due to nutritionally poor forage or seasonal dearth [5]. Beekeepers often use pollen substitutes to aid colonies through sparse periods or to prepare them for a spring buildup. These substitutes are largely composed of protein supplements and are most often prepared from crude protein isolates such as whey, soy, or yeast extracts [6]. Diets with these bulk nutrients may provide a great deal of protein, but they vary widely in other characteristics such as lipid, amino acid, or phenolic compound profiles [7, 8]. And importantly, these industrial protein sources lack the nutritional complexity of pollen grains.

Forager bees collect pollen from flowering plants and bring it to the hive where it is stored in comb cells near the brood chamber. This collected pollen is combined with nectar and glandular secretions from workers and stored as “bee bread.” Bee bread is consumed by young nurse workers to meet their own nutritional needs and to produce proteinaceous secretions from their hypopharyngeal glands to feed developing brood [9]. As bees age they transition away from consuming pollen to subsisting solely on nectar and its concentrated form, honey [10, 11]. These transitions are regulated by key developmental molecules, including juvenile hormone (JH) and the major lipoprotein vitellogenin (Vg) [12]. Vg expression has multiple influences on bee development, health, social immunity, and social regulation at both the individual and colony levels [13–15]. It is often used as a biomarker in assessing nutritional status in surveys of workers or hives [8, 16]. Vg levels operate in a double repressor model with those of JH to usher in transitions of bees from nurse to forager or to a long-lived overwintering stage [13, 17].

After ingestion by nurse workers, pollen grains are lysed in the midgut where the nutrients of the cytoplasm are rapidly absorbed [10, 18]. The refractory components of the spent pollenkitt accrue in the bee’s hindgut (the ileum and rectum) and provide substrates that the resident microbes of this compartment continue to digest [19, 20]. The matrix of indigestible sporopollenin fragments from lysed pollen grains is attached to the material of the intine layer, which contains a wide range of monosaccharides and polysaccharides including cellulose, hemicellulose, and pectin, as well as secondary plant compounds such as polyphenols.

The microbiota that inhabits the honey bee hindgut consists of a specialized group of core taxa along with several other more sporadic members [21]. The ileum, analogous to the small intestine in mammal guts, is coated by a thick biofilm of the proteobacterial associate *Snodgrassella alvi* and at least two *Gilliamella* species (*G*. *apis* and *G*. *apicola*). In contrast, the rectal community is made up mostly of Gram-positive clades of Lactobacilli (*Lactobacillus* nr. *melliventris* and *Bombilactobacillus* spp. formerly Firm-5 and Firm-4 respectively) and multiple *Bifidobacterium* species [22–24]. These bacteria, and even strains within these bacterial clades, have different sets of digestive enzymes that metabolize specific components of the pollen coat. For example, most *G*. *apicola* strains possess pectin lyase genes that degrade pectin, whereas *G*. *api*s does not [25–27]. *Bifidobacterium* species have variable numbers of glycoside hydrolase (GH) enzymes from different GH families that are able to digest distinct polysaccharides [27]. The presence of this bee gut microbiota has been previously linked to the proper development of immune function [28–30], developmental weight gain, and nutritional profile in workers [19, 20].

In this study, we carried out experiments to elucidate how an artificial protein-rich diet lacking pollen impacts the size and composition of the gut microbiome, the expression of key developmental genes, and the workers’ susceptibility to an opportunistic bacterial pathogen.

## Materials and methods

### Effect of diet on gut microbiota and on developmental gene expression

The same experiment was replicated in two locations and seasons: the University of Texas at Austin in July of 2020 (UT), and Texas A&M University (TAMU) in College Station, TX in October of 2020. We used field source colonies from which we removed brood frames containing late-stage pupae and placed them in frame cages inside incubators kept overnight at 35°C and 85% relative humidity (RH). We selected brood frames without floral food sources to ensure that the emerging bees would not have access to any food prior to beginning the experiment. The following morning, we collected hundreds of emerged workers and placed 30 of them in each of 5 cup cages belonging to 3 feeding conditions: control pollen-fed bees (**POL**), bees fed on the experimental artificial diet alone (**AD**), or bees that were fed a diet with hemicellulose and pectin components added (**ADA**). All cup cages were also provided with a sterile sucrose solution for bees to feed *ad libitum*.

We inoculated these <24-hour old bees with bacteria from adult nurse guts harvested from the source hives from which we obtained the emerged bees. We used a method similar to previous studies [31, 32] but added a filtration step to lessen the chance of residual pollen components being fed to the study groups. We prepared this inoculum by dissecting the hind guts from 50 nurses and homogenizing them with pestles in phosphate buffered saline (PBS) solution. The total volume of the homogenate and rinsate from pestles and tubes was brought to 10 mL. We briefly vortexed and then centrifuged this suspension for 1 min at 3,000 x g to pellet out large pollen grains. We then filtered the supernatant progressively through 100 μm, 70 μm, and 40 μm nylon filters (Millipore, Burlington, MA, USA). We added sterile sucrose syrup to the resulting filtrate and used 250 μL to coat the bolus of protein in the feeders of all cup cages. We stored the cup cages of inoculated bees at 35°C 85% RH for the remainder of the experiment. Additional protein diet (pollen or experimental) was added daily to all cup cages, as needed. Cups containing diets were replaced every two days and weighed to measure consumption for the TAMU trial. Cups were allowed to reach ambient humidity levels in the laboratory for 24 h prior to being weighed before and after consumption allowing the food to equilibrate to ambient room humidity levels as in [33]. We also assessed mortality and removed any dead bees daily. We collected 10 bees from each cage on days 7 and 14 after inoculation and placed them at -80°C until processing nucleic acids. We concluded the experiment 14 days after the cups were established.

**Fig 1:**
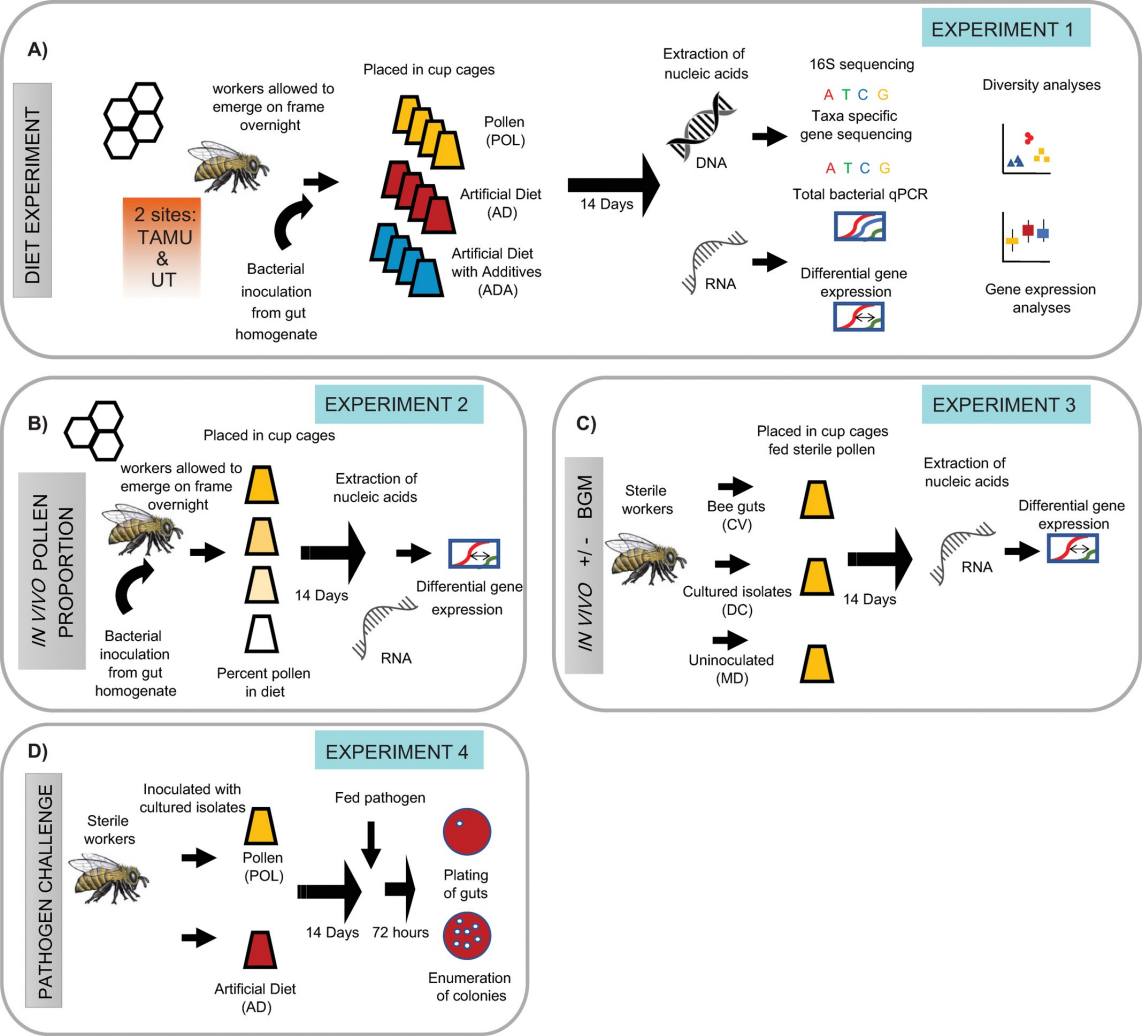
Design of experiments on effects of microbiota and diet. (A) *Effect of diet on the bee gut microbiota and gene expression*. We allowed worker bees to emerge from a frame overnight and inoculated them with conventional honey bee gut microbiota. We placed them into cup cages and fed them either pollen (POL), an artificial diet (AD), or an artificial diet that included hemicellulose and pectin additives (ADA). After 14 days we dissected the bees’ abdomens and extracted DNA and RNA from 7 workers sampled from 4 cups per condition per site. We used these nucleic acids to perform 16S rRNA gene or taxon-specific gene sequencing, as well as quantitative polymerase chain reaction (qPCR) of total copies of 16S rRNA genes. We used RNA to make cDNA and used qPCR to examine transcript abundance of developmental and dietary genes. (B) *Effect of proportion pollen in diet on expression of developmental genes*. We allowed bees to emerge on a brood frame overnight and then conventionalized them by feeding them a gut homogenate prepared from adult workers. We placed the bees into cup cages and fed each cup a different proportion of pollen mixed with an artificial diet (percentage of pollen in cups = 100%, 50%, 25%, or 0%). After 14 days we dissected the bees’ abdomens and performed RNA extraction and developmental gene assays as above (n = 8 bees per condition). (C) *Effect of microbiota on expression of developmental genes*. We pulled late-stage pupae from brood frames and allowed them to emerge as adults in sterile conditions. These sterile workers were either inoculated with gut homogenate from adult bees (conventionalized = CV), with co-cultured gut bacterial isolates (defined community = DC), or kept uninoculated (microbiota deficient = MD). We placed the bees into cup cages with sterile pollen and sugar syrup for 14 days. We then dissected abdomens, extracted RNA, and used this to synthesize cDNA and run qPCR to examine developmental gene expression. This experiment was performed twice because the CV group died in the first round (n = 8 bees from 2 cups per condition, repeated twice). (D) *Effect of diet on susceptibility to a bacterial pathogen*. We generated sterile workers as in (C), placed 20 workers into 2 cups, and inoculated them with a co-cultured defined community of gut bacteria. We fed pollen to one of the cups and an artificial diet to the other one. After 14 days we fed each of the bees 5 μL from and OD600 = 1 suspension of *Serratia marcescens* (strain KZ11) tagged with a kanamycin-resistant marker. We maintained these bees for 72 h, dissected their guts, and then plated dilutions of the homogenate on kan+ plates. We counted the number of resultant colonies (n = 15 bees per condition).

For the pollen source we used irradiated monofloral rape (*Brassica rapa*) pollen, as this is a monoculture frequently visited by *A*. *mellifera* foragers. Full details of the nutrient composition of the *Brassica* pollen used in this study can be found in Lau et al. (2022) [34]. For the experimental diet we used a formulation that simulated the macronutrient profile of the rape pollen with a 30:20 ratio of protein to lipids mixed with 50% sucrose, cellulose, and vitamins (the recipe is provided in S1 File). For the formulation of the artificial diet with additives (ADA) we added 100 mg of each of the following components per kg of total diet (to simulate hemicellulose components): beta glucan, arabinan, pectic galactan, and xyloglucan. Finally, to add the pectin backbone, we used polygalacturonic acid (Sigma, St. Louis, MO, USA). We stored all diets at -20°C prior to use. The experimental setup is summarized in Fig 1A, and sample information is included in S1 Table.

### Effect of proportion of pollen on developmental gene expression

We pulled late-stage pupae (with dark eyes but not yet moving) from frames and allowed them to eclose in sterile arenas as done in previous experiments [31]. Within 24 h after eclosure these microbiota-free, newly emerged workers were divided into groups of 25 bees in 4 cups. We provided each cup with a diet consisting of different proportions of *Brassica* pollen and an artificial diet: a) 100% pollen / 0% artificial diet, b) 50% pollen / 50% artificial diet, c) 25% pollen / 75% artificial diet, or d) 0% pollen / 100% artificial diet. We inoculated the pollen / diet mixtures with macerated bee guts in PBS, as above. We provided bees a sterile sucrose solution *ad libitum* and maintained the cup cages at 35°C and 85% RH for 14 days. We placed the bees at -80°C prior to dissection and RNA isolation from heads and abdomens. See Fig 1B for a summary.

### Effect of microbiota on developmental gene expression

We conducted two trials to examine the influence of the bees’ gut microbiome on the maintenance of vitellogenin and juvenile hormone esterase levels (see Fig 1C). To do this, we pulled bee pupae and allowed them to emerge as with the microbiota-effect experiment above. We divided these microbiota-free newly emerged workers into groups of 25 and placed them in 3 cups provided with sterile multifloral pollen and sucrose solution. To produce bees with a conventionalized (CV) microbiota, we placed in a cage a homogenate of hindguts from 3 hive workers disrupted with a pestle in 300 μL PBS over the pollen. In another cage we used 300 μL of a PBS suspension of a defined community (DC) group that we scraped from a Columbia Blood Agar (CBA) plate to an optical density at 600 nm of ~3. This defined community contained co-cultured isolates of *Gilliamella* (wkB1, wkB7), *Snodgrassella* (wkB2), *Bifidobacteria* (LCep5), and *Lactobacillus melliventris* (wkB8 and wkB10). The third cup was not inoculated and was used as a microbiota deficient (MD) control group. We maintained the cup cages at 35°C and 85% RH for 14 days and placed them at -80°C prior to dissection and RNA isolation from heads and abdomens. In the first trial all bees in the CV group died, so we repeated the experiment.

### Nucleic acid preparation for characterizing microbiota and developmental gene expression

We prepared 7 samples from 4 cups per each of the two sites (UT and TAMU) for each of the 3 feeding conditions (POL, AD, ADA). We first thawed bees on ice in order to prepare nucleic acids from experimental samples. We then dissected their abdomens and placed them in clean pestle tubes with 600 μL of RNA lysis buffer from the Zymo Quick RNA Miniprep kit (Zymo, Irvine, CA, USA). We homogenized the abdomens for 30 s and then placed this mixture into bead tubes containing 500 μL of 0.1 mm silica zirconia beads (Biospec, Bartlesville, OK, USA). We bead beat the samples for 1 min and then used the Zymo kit as per the manufacturer’s instructions to prepare 100 μL of RNA. We isolated DNA from these same samples by retaining the gDNA elimination column from the RNA preparations. We then washed the DNA columns with 500 μL of Qiagen wash 1 and 2 buffers from the Qiagen Dneasy kit (Qiagen, Germantown, MD, USA) and then eluted the DNA with 100 μL of molecular grade water.

We quantified and tested the RNA for purity by using a Nanodrop Lite spectrophotometer (Thermo, Waltham, MA, USA) and adjusted all RNA samples to a concentration of 25 ng/μL. We used 50 ng of RNA to generate cDNA using a Takara PrimeScript kit (Takara Bio Inc, Shiga, Japan). We diluted the resulting cDNA tenfold and used 1 μL per reaction for subsequent triplicate quantitative polymerase chain reactions (qPCR). This purification and cDNA synthesis strategy was used for extracting RNA alone from heads and abdomens with both the pollen-percentage feeding experiment and the microbial presence/absence experiment. We quantified DNA with a Qubit dsDNA broad range kit and diluted it one hundred-fold for subsequent endpoint and quantitative PCR.

### Use of metabarcoding to estimate relative community composition of microbiota

We performed metabarcoding of the V4 region of the 16S rRNA gene by using techniques and primers similar to those used in many studies of overall bacterial community composition and by following the specifics of previous metabarcoding libraries from our laboratory [35–37]. Additional information is summarized in the S1 File. To examine strain variation within *Giliamella* (using the gene *rimM*) and *Bifidobacterium* (using the *groEL* gene) lineages we used a taxon-specific single-copy gene strategy similar to other studies [37–39]. In both schemes, we amplified the target gene of interest in an initial PCR reaction, cleaned the amplicon with magnetic beads (Axygen, Union City, CA, USA), and then added Illumina specific adapters (Illumina Nextera dual indexes) and a barcode in a second PCR. We cleaned the resultant amplicon and sequenced it with an iSeq100 for the 16S rRNA gene samples and a miSeq for the single-copy gene analysis (Illumina, Hayward, CA, USA). Primer sequences, reaction conditions, and sequencing information is included in S2 Table.

### Processing and analysis of sequence reads from metabarcoding

We analyzed amplicon sequence data with Qiime2, including steps for quality control and read processing, resulting in compositional and diversity analyses (See S1 File for detail). We performed analyses on the experiments at the two sites separately, as there was enough compositional difference between communities at the sites to warrant this (*i*.*e*., Beta diversity showed a significant difference when looking between sampling sites). We examined differences between feeding groups at each site by looking at alpha diversity in samples (*e*.*g*., calculating Effective Species Number from Shannon Index values [40], phylogenetic diversity in samples, as well as species evenness [41]). We compared differences between groups by using Kruskal-Wallis rank sum tests and post-hoc multiple comparisons tests in R with the kruskal.mc command from the *pgirmess* package [42] and Tukey and Kramer (Nemenyi) test with Tukey distribution approximation for independent samples from the R package PMCMR [43]. We plotted charts in R using *ggplot2* [44]. We compared beta diversity between groups by using weighted UniFrac distance matrices and testing for significance with PERMANOVA tests.

We used the relative abundances found by analyzing groups of taxa in amplicon sequence variant (ASV) tables to calculate the absolute abundance of bacterial lineages. We divided the total number of 16S rRNA gene copies estimated from qPCR by the relative abundance proportions for each taxon and corrected for rRNA operon number per genome as done in [32]. These data are included in S1 Table. For statistical analysis and representation purposes, we added the number one to all entries in the absolute abundance table. We did this as many lineages had entries for samples with zero absolute abundance and these were not mathematically defined in logorithmic analysis. We then compared the absolute abundance of each lineage with Kruskal-Wallis rank sum tests and post hoc tests as mentioned above.

For the taxon-specific single-copy gene experiments to analyze strain dynamics within *Gilliamella* and *Bifidobacterium* lineages we used high throughput reads from the single-copy gene targets to look at effective species number within groups. We then performed an absolute abundance analysis by using relative proportion amounts to split reads from the qPCR/16S analysis that were associated with those taxa. Details on how this type of assay and analysis were performed are provided in the S1 File.

### Use of qPCR to quantify total bacterial abundance

We assessed total 16S rRNA gene copy number by using qPCR to quantify absolute numbers of copies in 1 μL of isolated gut DNA using previously described methods [31]. Briefly, we used dilutions of a defined concentration of the plasmid pGemT bearing the target segment of the 16S rRNA gene as a standard curve. We used the primers 27F/355R to amplify this sequence in triplicate reactions of all samples and used the standard curve to obtain the copy number concentration. We corrected these counts for dilution. We used these total abundance values to calculate absolute abundance as described above.

### Use of qPCR to examine expression of developmental genes

For qPCR we used triplicate reactions of 1 μL of a 1-to-10 dilution of cDNA generated from 50 ng RNA. This was used to examine differences in gene expression for the developmental genes vitellogenin (*Vg*) and Juvenile Hormone Esterase (*JHE*) [45] relative to the housekeeping gene *RPS5a* [46]. We also tested subsets of samples from the diet-effect feeding experiment and the microbiota-effect feeding experiment with and without microbiota for the Insulin receptor genes *AmInR1* and *AmInR2* [20]. We have included the qPCR primer sequences and amplification programs used in S2 Table.

To analyze relative gene expression we used the 2^-ΔΔ*CT*^ [47] method and investigated fold-change differences between control and treatment groups. We first log_2_ transformed 2^-ΔΔ*CT*^ values and then analyzed datasets for normality by looking at quartile to quartile histograms and also by testing with the Shapiro-Wilk test for normality. Because some sets failed to have normal distribution and some had small numbers of replicates and could not be adequately assessed we used nonparametric tests to examine groups for statistical difference. For tests between two groups, we used the Mann-Whitney-Wilcoxon rank sum test and for three group tests we used Kruskal-Wallis with post hoc tests as mentioned before. Only significant interactions between control (DC in the first trial and CV in the second) and experimental groups are reported.

### Effect of diet on pathogen susceptibility

We allowed bees to emerge from a brood frame and placed them in cup cages with either monofloral pollen or the experimental diet alone (diet AD). We then inoculated them with filtered nurse gut homogenate, as above. We maintained each group in a cup cage for 5 days then fed them each 5 μL of OD600 ~1 of kanamycin resistant *S*. *marcescens* KZ11 (a strain originally isolated from bees [48]) and modified via Tn5 integration [49]. After 72 h we removed and homogenized their guts in 100 μL PBS. We then performed 10-fold dilutions and spotted 10 μL per dilution onto CBA plates with kanamycin at 50 μg/mL. We enumerated colonies in order to gauge the level of infection after overnight incubation at 35°C. See the experimental design in Fig 1D. We chose this organism as it is a described opportunistic pathogen of honeybees that appears to be linked to gut dysbiosis, is experimentally tractable and that can cause mortality in adult workers [24, 48–50].

## Results

### Overall diet consumption decreased over time for all groups, but the rate of decrease was significant in POL bees compared to the AD and ADA groups (S1 Fig) (experiment 1 summarized in Fig 1A)

As a consequence of the slowing of consumption, POL bees consumed less food over the course of the experiment (40.1 mg ± 2.2 mg SD) and significantly less than AD bees (47.8 mg ± 3.1 mg SD) (S2 Fig). Survival was high throughout each treatment group with all diets having greater than 89% of individuals alive at the end of the experiment (S3 Fig).

### The overall size of the gut microbiota increased with the use of the artificial diet

**Fig 2:**
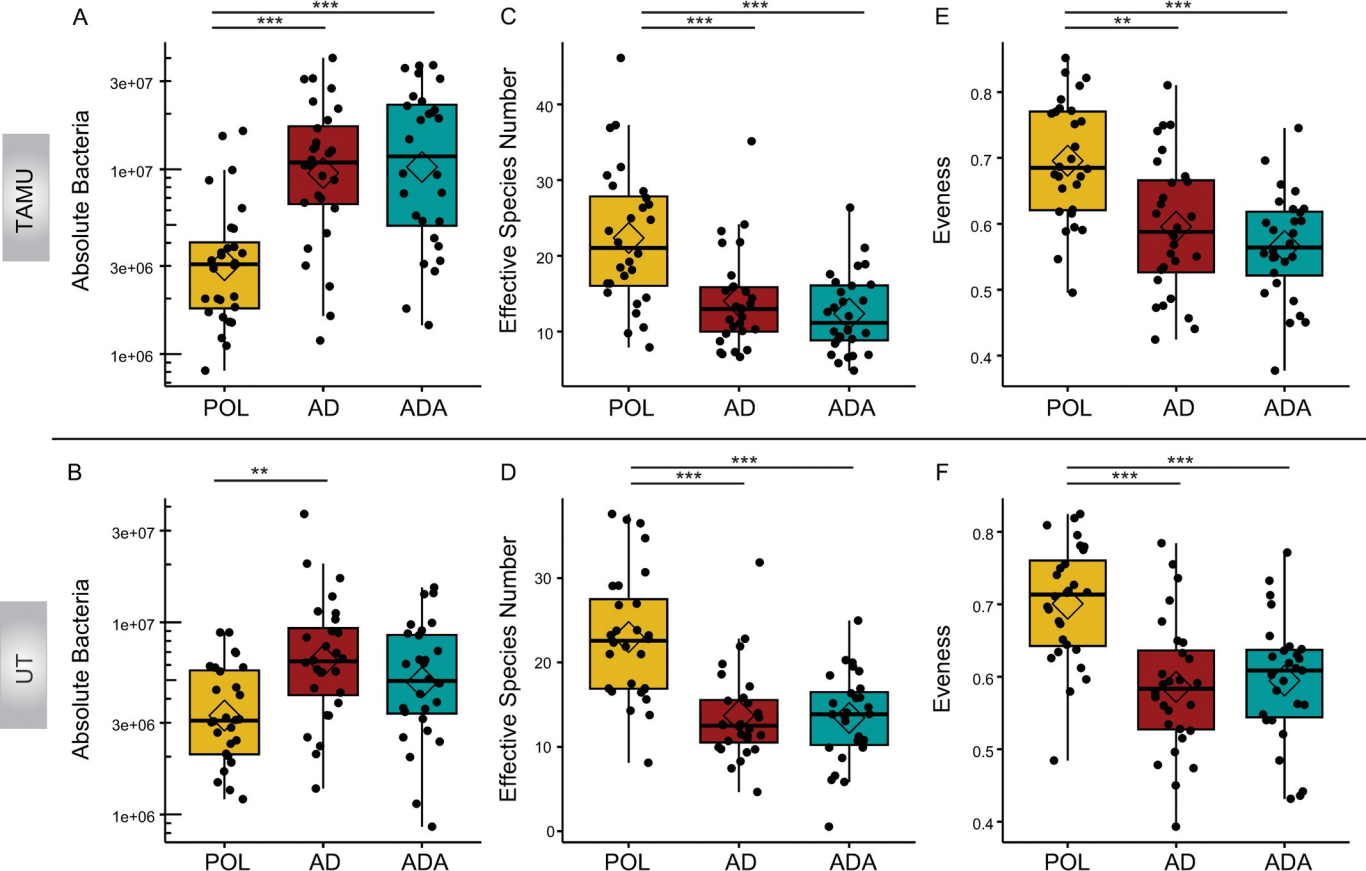
Dietary effects on microbiota. Effects of bees being fed pollen (POL) versus an artificial diet (AD) or an artificial diet with additives (ADA) on the gut microbiota at 2 sites (TAMU and UT, n = 28 bees per condition per site). (A-B) Plots of the absolute abundance of total gut bacteria per 1 μL bee gut DNA. Total gut absolute abundance is the summed values of absolute abundance from all taxa in a sample. Total 16S rRNA gene copies were estimated by qPCR split by taxa relative abundance and corrected for rRNA operon number per genome. (C-D) Plots of species richness as assessed by effective species number. (E-F) Plots of species evenness as demonstrated by Pielou’s evenness index. *p < 0.05, **p < 0.01, ***p < 0.001, post hoc pairwise comparisons using Tukey and Kramer (Nemenyi) test with Tukey distribution approximation for independent samples following Kruskal-Wallis. Diamonds indicate the mean value for each boxplot in this and following figures.

For the experiment conducted at TAMU, POL bees had a smaller absolute bacterial population (POL absolute bacterial mean = 4.1e6 ± 4.2e5 SEM per μL) than either of the groups receiving the artificial diet (AD mean = 1.3e7 ± 4.2e5 SEM per μL, ADA = 1.5e7 ± 2.2e6 SEM per μL) (*H*(2) = 24.1, *P* = 5.9e-6, Kruskal-Wallis). In the UT experiment the POL group (mean = 3.8e6 ± 4.2e5 SEM per μL) microbiota was smaller than that of the AD group (mean = 8.2e6 ± 1.3e6 SEM per μL) (*H*(2) = 12.1, *P* = 0.0023, Kruskal-Wallis), but similar to that of the ADA bees (mean = 6.1e6 ± 7.4e5 SEM per μL) (Fig 2A).

### The use of a protein substitute resulted in gut microbiota with lower species diversity and evenness compared to the microbiota of pollen-fed bees

Bees in both the AD and the ADA groups had lower alpha diversity, as measured by effective species number (ESN), than POL bees (Fig 2B). This lowered diversity was significant in both the AD and ADA groups for both the TAMU (*H*(2) = 22.4, *P* = 1.4e-05, Kruskal-Wallis) and UT experiments (*H*(2) = 28.9, *P* = 5.3e-07, Kruskal-Wallis). POL fed groups had mean ESN values of 22.4 at TAMU and 23.0 at UT compared with values in the 12 to 14 range for artificial diet groups at both sites. Similarly, both artificial diet groups had significantly lower evenness indices (Pielou) than the POL bees at both sites (TAMU, *H*(2) = 22.4, *P* = 1.4e-05, Kruskal-Wallis) (UT, *H*(2) = 24.0, *P* = 6.2e-06, Kruskal-Wallis) (Fig 2C).

### Pollen-fed bees had higher populations of bacteria linked to floral or hive environments

**Fig 3:**
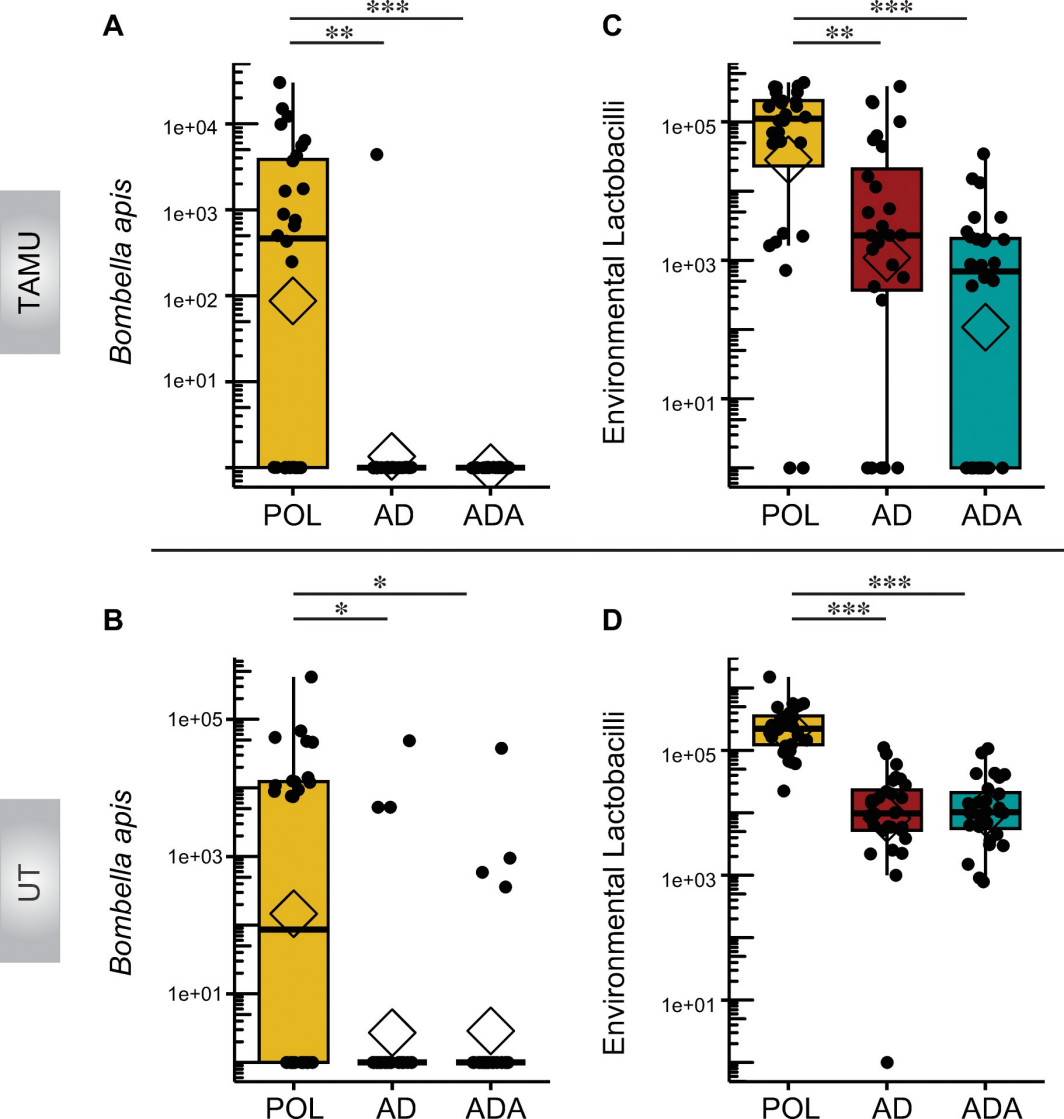
Effects of bees being fed pollen (POL) versus an artificial diet (AD), or an artificial diet with additives (ADA), on the absolute abundance of hive-associated bacterial taxa obtained from bee guts at TAMU and UT. (A-B) Plots of *Bombella* species absolute abundance. (C-D) Plots of environmental or hive-associated *Lactobacilli* absolute abundance. *p < 0.05, **p < 0.01, ***p < 0.001, post hoc pairwise comparisons using Tukey and Kramer (Nemenyi) test with Tukey distribution approximation for independent samples following Kruskal-Wallis.

POL group bees had larger populations of bacteria associated with pollen, nectar, and the hive environment, compared to bees that were fed artificial diets. Notably, *Bombella apis* (previously referred to as *Parasaccharibacter apium* and *Saccharibacter sp*., and in earlier literature as Alpha 2.2 [51, 52]) was significantly more abundant in POL bees at both sites (Fig 3A and 3B). POL bees at TAMU and UT had 16 and 14 samples out of 28 per site that had any *B*. *apis* whereas the experimental groups had a presence in only 1 sample at TAMU and 4 at UT along with 0 and 4 respectively for the ADA groups. The mean absolute abundance for *B*. *apis* was elevated in the POL groups at both sites with 3.4e+03 ± 1.3e+03 SEM per μL at TAMU and 2.6e+04 ± 1.5e+04 SEM per μL at UT compared to much lower populations for the experimental groups (TAMU, *H*(2) = 34.23, *P* = 3.69e-08, Kruskal-Wallis) (UT, *H*(2) = 16.3, *P* = 0.00029, Kruskal-Wallis). Similarly, POL bees had many more hive-associated or environmental Lactobacilli, composed mostly of *Apilactobacillus kunkeei* (Fig 3C and 3D), a species associated with diverse bee species, flowers, and the hive environment [53, 54]. Other environmentally associated bacteria had uneven distribution patterns between sites. For example, POL bees at the TAMU site had lower incidence of “other” bacteria (mostly *Streptococcus* and *Staphylococcus* associated ASVs) than bees fed on artificial diets (presence in 4 POL samples vs. 21 and 22 samples in the AD and ADA groups). Absolute abundance was sporadic but significantly less in the Pol group at TAMU than in either of the experimental groups (*H*(2) = 24.3, *P =* 5.2e-06, Kruskal-Wallis). POL bees at the UT site also had a lower occurrence of these lineages (presence in 9 samples vs. 20 and 13 in the AD and ADA groups) and had significantly lower absolute abundance than the AD diet (*H*(2) = 8.1, *P =* 0.0173, Kruskal-Wallis) (S4 Fig).

### Differences between bacterial absolute abundance for multiple core taxa were more pronounced at one site than the other

The POL group at TAMU and UT had significantly lower absolute abundances of multiple lineages than the AD fed group for taxa including *Bartonella apis*, *Bombilactobacillus* spp. (at TAMU only), *Lactobacillus melliventris*, and *Bifidobacterium* spp. The TAMU POL group was also lower than the ADA group for these taxa. Both sites had significantly lower amounts of *S*. *alvi* for bees in the POL group compared to bees fed the AD or ADA diets (S5A–S5J Fig).

### The proportion of samples with enough *Gilliamella* single-copy reads for analysis was lower in artificially fed bees

We based the analysis of proportions on a) whether a sample amplified at all with the *rimM* single-copy gene primers, or b) if it did amplify, if there were more than 500 *Gilliamella*-related reads remaining after removing *Frischella* read contamination. This read cutoff was determined as a result of alpha diversity analysis, which showed this to be the minimum read depth to capture sample diversity and maintain the maximum number of samples. Many samples dropped out in the AD and ADA groups at both sites, whereas most POL samples were retained. We analyzed these proportions with a one-sided Fisher’s exact test and found significantly lower proportions of samples with a sufficient number of reads or both experimental groups at TAMU (AD and ADA), as well as for bees in the AD group at UT (Table 1).

**Table 1:** Numbers of samples per treatment with sufficient *Gilliamella* rimM reads for analysis after quality control filtering (n = 14 at each site per feeding group).

Diet treatment	TAMU	UT
POL	12	12
AD	3*** (0.0009)	4** (0.0032)
ADA	4** (0.0032)	9 n.s.

These are samples that amplified with specific primers and had > 500 reads after filtering out off target sequences. *P*-values are shown in parentheses. One-sided Fisher’s exact tests

** *p* < 0.01

***p < 0.001.

### The absolute abundance and strain diversity of *Gilliamella* spp. were largely unchanged by diet

The estimates of the absolute abundance of all *Gilliamella*-related reads based on 16S rRNA gene amplicons were statistically similar between diets at both sites, with only the ADA diet showing a decrease at the TAMU site (S6A and S6B Fig). The power of the analyses based on *rimM* amplicons was low due to the few AD or ADA samples that generated sufficient reads. Effective species number was lower for the ADA group at the UT site (S6C and S6D Fig). Absolute numbers based on *rimM* did not differ for *G*. *apis* or *G*. *apicola* at either site (S6E and S6F Fig). These results are likely biased, potentially masking differences, as samples with fewer *Gilliamella* were dropped from the analysis.

### There were no significant differences in the effective species numbers of *Bifidobacterium* spp. between the pollen- and diet-fed groups at either site

Alpha diversity metrics for the *Bifidobacterium* single-copy gene analysis (*groEL*) did not reveal significant shifts in diversity between diets (S7 Fig), in spite of the observation that the experimental diet-fed bees had larger absolute abundances of *Bifidobacterium* spp. (S5G and S5H Fig).

### Transcripts of genes encoding Vitellogenin (*Vg*) and Juvenile Hormone Esterase (*JHE*) were much lower in bees that were fed an artificial diet compared to bees that were fed pollen

**Fig 4:**
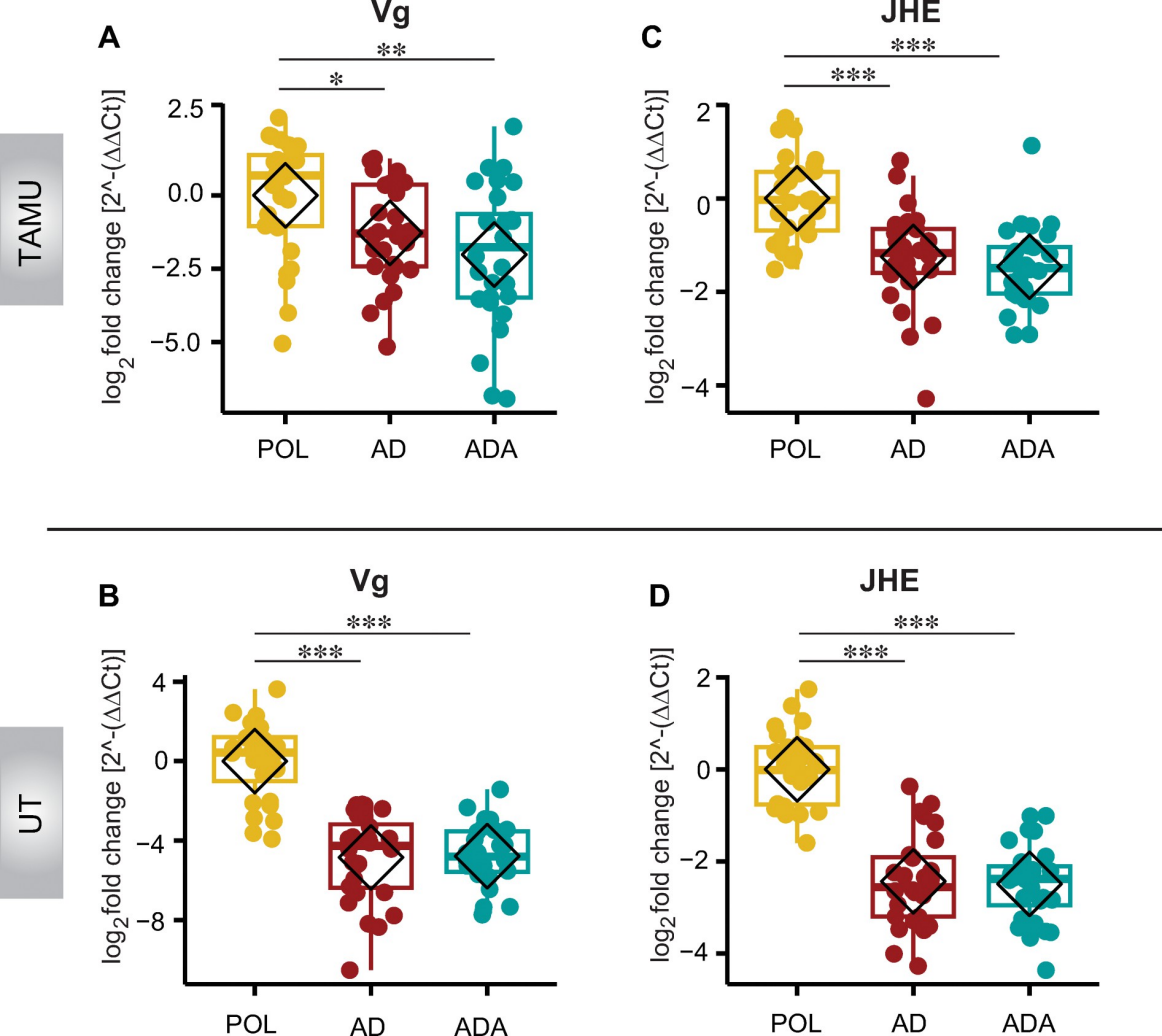
The effect of diet on developmental genes. The impact of honey bees being fed pollen (POL) versus an artificial diet (AD), or an artificial diet with additives (ADA) on developmental gene expression relative to the reference gene *RPS5a* in abdomens from bees at TAMU and UT. (A-B) Relative mRNA expression of the gene encoding Vitellogenin (*Vg*). (C-D) Relative mRNA expression of juvenile hormone esterase (*JHE*), a proxy for juvenile hormone; n = 7 bees from 4 cups per condition per site. *p < 0.05, **p < 0.01, ***p < 0.001, post hoc pairwise comparisons using Tukey and Kramer (Nemenyi) test with Tukey distribution approximation for independent samples following Kruskal-Wallis.

We examined the number of transcripts of *Vg* and *JHE* relative to the reference gene (*RPS5a*) in the abdomens of bees used in the initial diet feeding experiment. At both sites, transcripts for both genes were significantly lower in all groups of bees that were fed the artificial diets (Fig 4A–4D). It is interesting to note that *Vg* relative expression was much lower in experimental groups at the UT site when compared with TAMU samples. The UT groups had log _2_(2^-ΔΔ*CT*^) values of -4.85 ± 0.41SEM and -4.79 ± 0.30 SEM for AD and ADA groups compared with -1.28 ± 0.32 SEM and -2.02 ± 0.45 SEM for those groups at TAMU. Additional results of statistical tests for these samples may be found in S1 Table.

**Fig 5:**
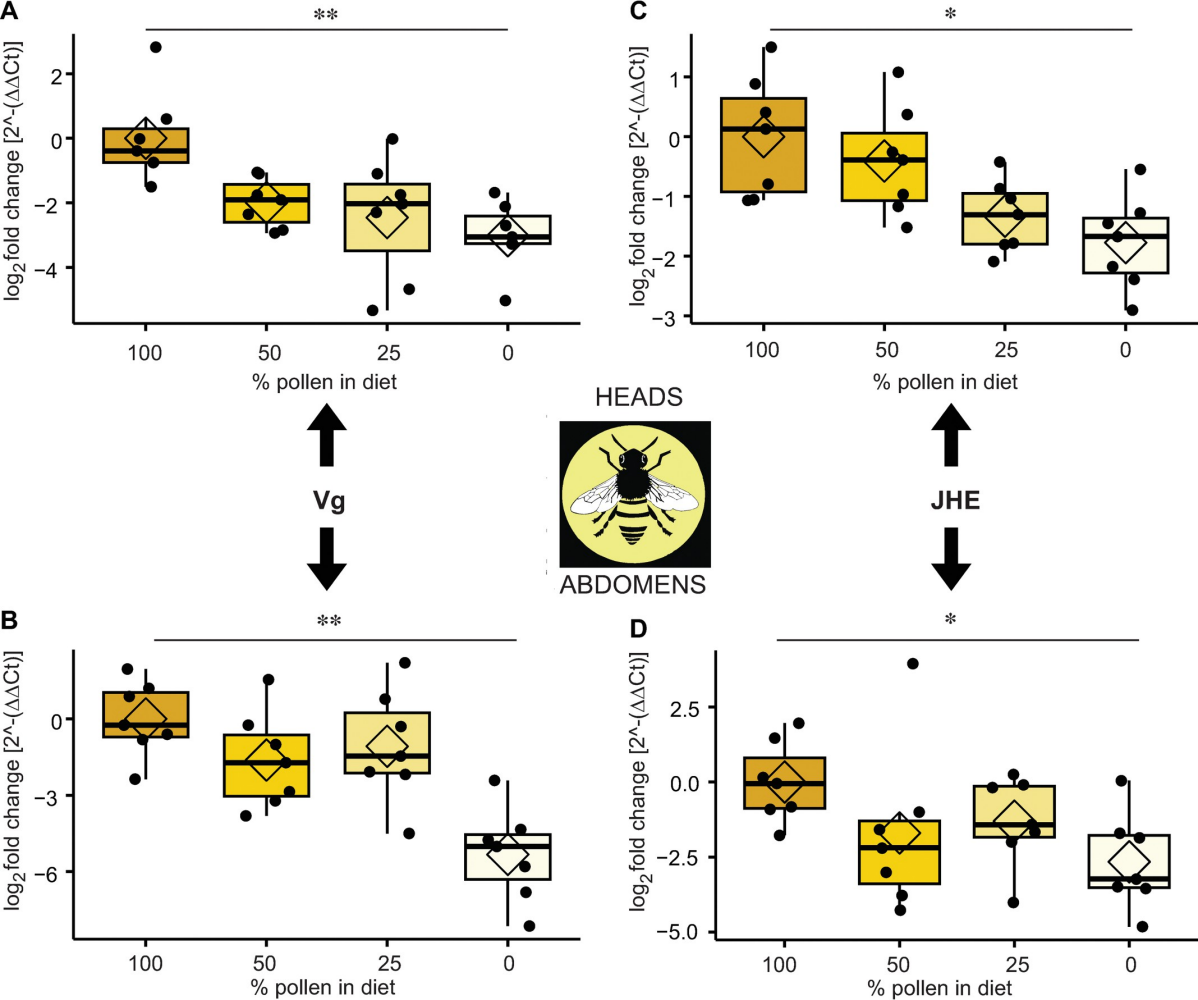
The effects of the proportion of pollen in the diet on the relative expression of developmental genes. Gene expression in heads and abdomens of 14-day-old honey bee workers with conventional gut microbiota. (A) *Vg* in heads. (B) *Vg* in abdomens. (C) *JHE* in heads. (D) *JHE* in abdomens (n = 7 samples per condition per tissue). *p < 0.05, **p < 0.01, ***p < 0.001, post hoc pairwise comparisons using Tukey and Kramer (Nemenyi) test with Tukey distribution approximation for independent samples following Kruskal-Wallis.

The experiment on the effect of percent dietary pollen (experiment 2 summarized in Fig 1B) on the expression of these genes in heads and abdomens validated this result. Expression of both of these genes in heads and abdomens follows a mostly stepwise downward trend paralleling the reduction of pollen in the diet. Comparisons between samples with 100% and 0% pollen are significantly different for both genes in both heads and abdomens (Fig 5A–5D).

### Experiments to examine the impact of gut bacteria on transcripts of Vitellogenin (*Vg*) and Juvenile Hormone Esterase (*JHE*) yielded ambiguous results (experiment 3 summarized in Fig 1C)

**Fig 6:**
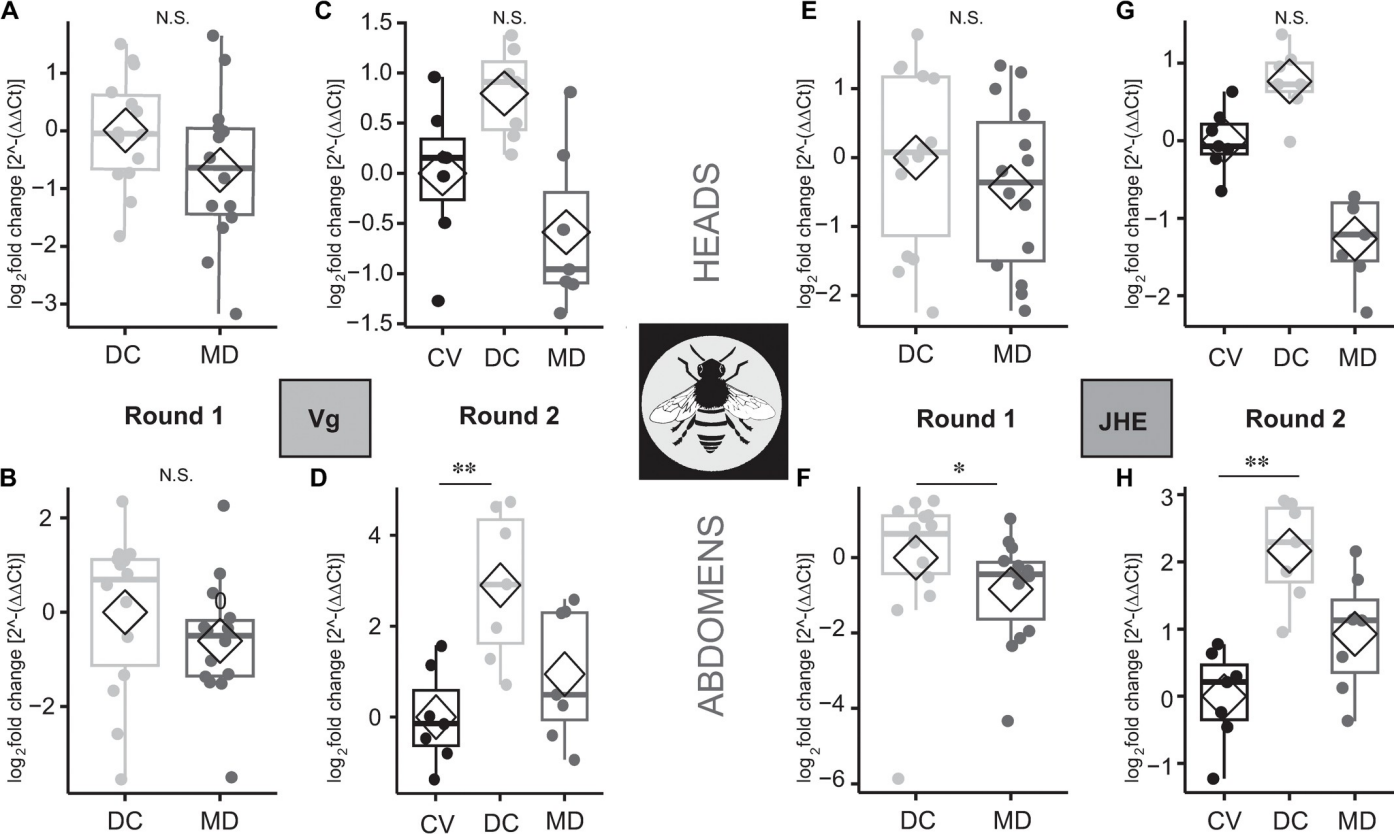
The effects of gut microbiota presence on the expression of developmental genes. *Vg* or *JHE* in the heads and abdomens of 14-day-old workers fed a pollen diet. MD = microbiota deficient bees, CV = bees colonized with conventional microbiota, DC = bees fed a defined community of cultured bee gut isolates. (A-B) The first experimental trial showing the relative expression of *Vg* in the heads and abdomens of DC and MD bees (n = 7 per condition per tissue). (C-D) The second experimental trial showing the relative expression of *Vg* in the heads and abdomens of CV, DC, and MD bees. (E-F) Relative expression of *JHE* from heads (in the first trial only) and abdomens of sampled bees. (G-H) Relative expression of *JHE* from heads (in the second trial only) and abdomens of sampled bees. Each boxplot represents n = 7 samples per condition per tissue. *p < 0.05, **p < 0.01, ***p < 0.001, Mann- Whitney Wilcoxon test for 2 category tests and for 3 category test: post hoc pairwise comparisons using Tukey and Kramer (Nemenyi) test with Tukey distribution approximation for independent samples following Kruskal-Wallis.

The bees conventionalized by being fed an adult gut homogenate died in the first microbiota presence/absence experiment, potentially due to the presence of a pathogen in the homogenate. Thus, this first experiment only included a cohort of bees fed a cultured defined community of gut isolates (DC) and microbiota-deficient (MD) bees. We used the DC group as the expression control group in this analysis. Although the microbiota-deficient bees trended towards lower expression levels for both genes, differences were not significant (Fig 6A, 6B and 6E) except for JHE relative expression in the abdominal samples (Fig 6F). The second experimental round, which included conventionalized bees as the expression control group, showed higher transcript levels in the DC group (though not significantly higher than the CV controls) for both *Vg* and *JHE* in the bees’ heads (Fig 6C and 6G) and abdomens (Fig 6D and 6H). CV bees had gene expression levels similar to MD bees for *Vg* and JHE and were significantly lower than DC bees in abdomen tissue (Fig 6G and 6H). Results of statistical comparisons may be found in S2 File.

### Bees without microbiota had higher insulin receptor gene transcript levels than those with a defined community

These differences were significant for both *AmInR1* and *AmInR2* for abdomen samples, and for *AmInR1* for head samples (S8 Fig).

### Insulin receptor gene relative expression data yielded different results between the UT and TAMU samples

Transcripts for *AmInR1* and *AmInR2* were significantly higher in AD fed groups at the UT site but did not demonstrate a significant difference in the TAMU samples (S9 Fig).

### Bees raised on a pollen diet were more resistant to infection by an opportunistic bacterial pathogen than those raised on the artificial diet (experiment 4 summarized in Fig 1D)

**Fig 7:**
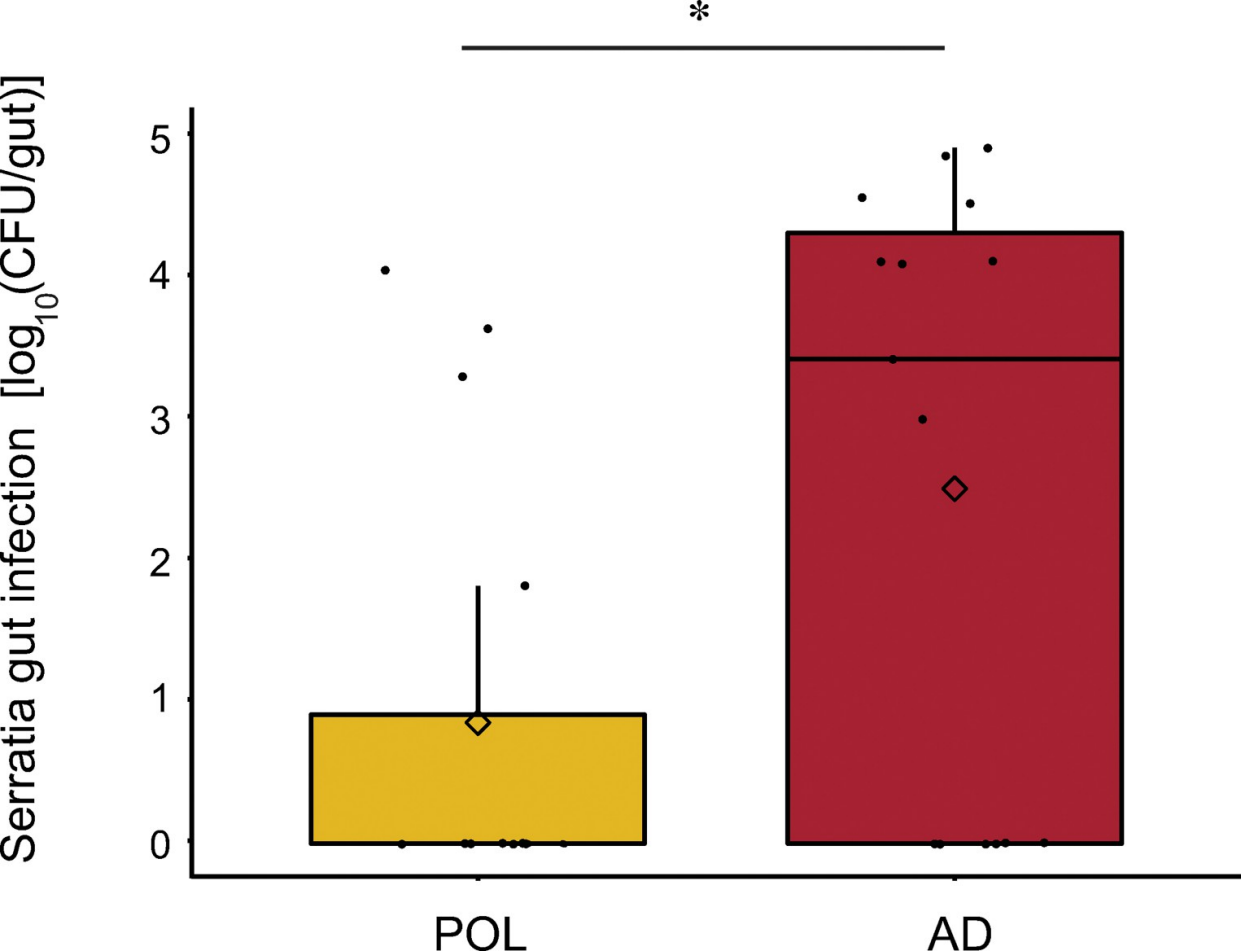
Effect of diet on an opportunistic pathogen. Boxplots showing the log_10_ CFU of the bacterial pathogen *Serratia marcescens* strain KZ11 (modified for kanamycin resistance) per plated bee gut obtained from bees with conventional microbiota that were fed either pollen (POL) or an artificial diet (AD). Guts were plated 72 h after 14-day old adults were orally inoculated with 5 μL of OD_600_ = 1 bacterial suspension (n = 15 per group). *, *p* < 0.05, Mann-Whitney Wilcoxon.

We measured abundance of the pathogen *Serratia marcescens* KZ11 (labeled with a Kan resistant marker) 72 h after infection of 14-day-old bees fed on pollen versus an artificial diet. We recovered much higher pathogen levels from bees fed the artificial diet (Fig 7). While many samples in both groups had no infection at all, the AD group bees had more samples with viable levels of KZ11 (9 of 15 were positive for AD, versus 4 of 15 being positive for POL) and higher concentrations of KZ11 in the AD group than those in the pollen group (W = 62, *p* = 0.02187, Mann-Whitney Wilcoxon). Since we used a single cup cage of bees per feeding condition and because bees frequently share food, bees within each group are not independent.

## Discussion

Our study showed that providing honey bees a pollen substitute, even one formulated to match the macronutrient profile of a natural pollen source, caused minor changes in the bee gut microbiome composition, and much larger changes in the expression of regulatory and developmental genes and in the ability to suppress an opportunistic pathogen.

Studies in many systems show that diet can have major impacts on gut microbiome composition, and specifically that diets with a diverse array of plant polysaccharides favor more diverse gut microbiomes [55]. This effect is best documented in mammalian hosts. For example, woodrats fed more diverse plant diets show higher gut microbiome diversity [56]. Also, a large body of research on the human microbiome shows that adding dietary fiber results in an increased in community diversity and in anti-inflammatory metabolites) [57]. In our study, the artificial diet is expected to contain fewer types of plant polysaccharides, and it did result in lower overall microbiota diversity, as assessed by effective species number and reduced taxonomic evenness of communities. Not surprisingly, we observed higher absolute abundance of pollen-associated bacterial species in pollen-fed bees compared to bees fed the experimental diets. These results accord with findings that adding phytochemicals to the sucrose solution provided to bees results in higher gut community diversity [58]. Such phytochemicals are found in pollen, but are lacking in the artificial diets we provided to bees.

In the main feeding experiment, results on microbiome composition differed somewhat between the two sites. For diet-associated differences in abundance of individual bacterial taxa, TAMU site samples generally showed larger differences between pollen and artificial diet groups than UT site samples. These site differences could reflect differences in initial microbiota composition, host condition due to environmental differences, or host genetics, as the bees at both sites came from different sources. The experiments at UT were conducted in July whereas those at TAMU took place in October so seasonal fluctuations in the microbiome or bee condition may have also played a role [59, 60]. That said, many observations were consistent between sites.

Regarding how diets impact host expression of developmental genes, pollen-fed bees showed much higher expression of *Vg* and *JHE* in abdomens as compared to bees fed either artificial diet. This effect was observed at both sites. Both vitellogenin and juvenile hormone have been demonstrated to regulate behavior in social insects [13, 61]. In normal bee development, *Vg* levels increase during the first week or two of life and then diminish as JH levels begin to rise [17]. These changes usher in a transition in worker development from being hive-bound to foraging. This developmental shift is reflected in our consumption data, as bees expectedly decreased their dietary consumption over time.

Interestingly, bees given the artificial diets consumed more diet over time compared to bees given natural pollen. There are several potential explanations for this observation. First, bees given the artificial diets may increase their dietary consumption to meet thresholds for other nutrients present in pollen but deficient in artificial diets [7, 62]. Bees given the natural pollen diet consumed more diet earlier in the experiment and may have sufficiently met their nutritional needs within the first few days. Conversely, bees might restrict consumption to prevent a nutrient from exceeding a particular threshold. For example high levels of potassium are present in the *Brassica* pollen used in this study [34]. Another potential explanation is that pollen and artificial diets differ in digestibility. Bees may only be half as effective when digesting the protein from artificial diets compared to natural pollen [63, 64], requiring individuals to consume more artificial diet to meet similar thresholds to pollen. Potentially, the diets lost mass at different rates to differences in evaporation rate. To address this, we equilibrated the feeding cups to ambient humidity before feeding and weighing, but differential evaporation remains a potential issue [33].

Both *Vg*, encoding vitellogenin, and *JHE*, encoding juvenile hormone esterase, were expressed more in bees fed pollen. Vitellogenin is produced in the abdominal fat bodies, while JH is produced in the corpora allata, located behind the brain [65], but this hormone circulates and has effects throughout the entire body [66]. *JHE*, which regulates levels of circulating *JH*, is expressed in fat body in the abdomen [45, 67]. The diminished *Vg* transcripts in bees that were fed pollen substitutes has been observed previously [8, 68, 69], but the parallel reduction in *JHE* expression is a novel result. Because we did not directly measure JH, the effects of diet on JH titer are not clear, but the substantial reduction in *JHE* transcripts in bees fed the artificial diet suggests that diet does have downstream implications for the circulating levels of JH in the hemolymph.

A previous study of vitellogenin and JH titers in the hemolymph of Africanized honey bees [70] found no effect on JH levels when foragers were fed pollen substitutes compared to those that were fed a pollen diet. However, that study only followed workers through day 6 of their adult life, and JH levels typically increase later in development, closer to day 15+ post emergence [17]. It remains unclear whether lack of pollen in a developing worker’s diet changes the timing of the transition from nurse to forager, as reflected in the age of first flight, hypopharyngeal gland size in nurse bees, gustatory responses, fat body metabolism, hematocyte formation [71], or foraging behavior [17].

Vitellogenin has also been implicated in mechanisms for transgenerational immune priming within *A*. *mellifera* colonies [72]. For instance, nurse bees are exposed to bacterial pathogens during their hive-related duties, whereby fragments of cellular components of these bacteria become attached to vitellogenin. These immunological complexes are then fed to the queen, and the immune systems of her developing eggs are primed against these signals. In this manner, reduced levels of vitellogenin could result in wide-ranging, long-term immune deficiencies within the colony as a whole, not simply within the individual worker.

In our experiments, higher proportions of dietary pollen in the diet resulted in increased expression of *Vg* and *JHE* in both heads and abdomens. It is unclear whether the presence of the core gut microbiota contributes to this pattern. A previous study found large differences in *Vg* transcript levels between CV and MD bees at 7 days of age [20], whereas we found no significant differences in *Vg* levels between CV and MD bees. However, the bees in our study were assayed at 14 days of age, and the differences observed in [20] could be lost as the adult bee ages. Furthermore, the introduction of microbes via uncharacterized fecal transplants (*i*.*e*., CV bees) can introduce potential variability, including the possibility of inadvertently exposing transplant recipients to pathogens. Regarding nutrition, using artificial diets that lack a limiting nutritional component (*e*.*g*., a particular amino acid) may interfere with the regulation of this hormone (JH) or lipoprotein (Vg). A previous study looking at the use of spirulina as a prebiotic for bee diets [73] found that bees fed sucrose only had much lower levels of Vg than those reared on pollen. The same study also found that bees raised on a commercial pollen substitute had higher levels of Vg compared to pollen-fed bees, contrary to our results. The product was not specified, raising the possibility that pollen substitutes differ in effects on expression of developmental genes.

The presence of the gut microbiota also appears to influence expression of insulin receptor genes. In an earlier study, the presence of a microbial community raised the transcription of insulin receptor genes *AmInR1* and *AmInR2* in the abdomen in bees sampled at 7 days of age [20]. Conversely, our study, which sampled bees at day 14 of age, showed lower transcript levels of the insulin receptor genes *AmInR1* and *AmInR2* in the abdomen of bees that had a defined bacterial community compared to microbiota deficient bees (S9 Fig). This difference may be related to the transition of bees from performing in-hive tasks to foraging when higher levels of *InR1* and *InR2* are frequently observed [74]. Potentially this observation signals that bees that lack a standard microbiota transition to foraging roles sooner than normal. We also observed in our initial dietary feeding study that the levels of receptor gene transcripts were lower in the pollen-fed group (significantly at the UT site) versus those fed on artificial diets (S8 Fig), potentially reflecting a perturbed development schedule that is influenced by nutrition.

Our experiments on susceptibility to an opportunistic bacterial pathogen suggest that artificial diets may interfere with host immune capabilities. In an inoculation and recovery experiment with bees provided a conventional gut microbiota, we found that fewer viable cells of the opportunistic bacterial pathogen *Serratia marcescens* strain KZ11 were recovered from pollen-fed bees than from bees fed the experimental diet three days after pathogen challenge. Although our artificial diets match the overall protein and lipid profile of the *Brassica* pollen we used, *Brassica* pollen contains a diverse and complex range of lipids and fatty acids, which may contribute to inhibiting the growth of pathogenic bacteria [75–77]. The physical structure of pollen grains may also play a role in reducing gut pathogen infection [78].

Interestingly, we did not see big diet-induced shifts in strain composition within *Gilliamella spp*. and *Bifidobacterium spp*., the two groups that we investigated with single-copy gene metabarcodes. Furthermore, we had anticipated seeing drops in the alpha diversity for both taxonomic groups in the AD groups. We anticipated a decrease in *G*. *apicola* abundance in the AD group, presumably due to the lack of pectin, with a recovery in the ADA group as complex ingredients were added back to the diet. Although we observed a small but significant drop in *Gilliamella* total alpha diversity at the UT site for the ADA group when examining the single-copy gene data (S3 Fig), other shifts were not apparent. The shifts in absolute abundance of the two *Gilliamella* species may have been obscured by a low number of useable observations (Table 1), especially at the TAMU site.

It is difficult to parse the individual contributions of nutrition and bacterial gut community on adult honey bee health, given that the two are intimately related [63, 79, 80]. The type and amount of nutrients accessed and processed by the bee host determines the substrates available to gut microbes [27, 81]. Similarly, processed metabolites from the gut microbiota have repercussions for host health [19]. Differences in diet have been linked to changes in the bee gut microbiota, with impacts on stored fat levels and host weight of adult workers [82]. In bee larvae, for example, microbe-free pollen results in lower mean weights compared to larvae that are fed colonized pollen [83]. Feeding bees artificial pollen substitutes or carbohydrates alone has been shown to change gene expression of developmental and immunity related genes [68]. Similarly, changes in bacterial composition or abundance have been shown to influence weight gain, developmental hormone expression [20, 69], immunity factors [28, 29, 69], and resistance to pathogens [32, 37, 49, 69]. An additive effect from the interplay of nutrition and microbiota likely has consequences for host health, but the effects of this interplay remain to be explored.

Unexpectedly, the addition of hemicellulose and pectin components to the artificial diet has no detectable effects on gut microbiota composition or gene expression. Perhaps we did not augment the artificial diet with enough of these components to elicit a response from the microbiota. Conversely, the added components may not have been sufficient to capture the substrate complexity of the pollen coat [84].

The finding that the composition of microbial communities was minimally affected by the absence of pollen components was unexpected. No core lineages were lost between pollen-fed and artificial diet-fed bees though total microbial diversity was appreciably lower in the two artificial diet treatments compared to the pollen diet treatment. Most of this lack of diversity came from non-core taxa typically associated with the hive or other environments, not the core gut microbiome. The expansion in the absolute size of the total community and of some of the lineages (as with *Bifidobacterium* spp. and *S*. *alvi*) in one or both sites is probably a result of the easily digestible components in the artificial diet, which were provided in enough excess to contribute substrate for bacteria in the hindgut compartments. These results, taken along with previous studies that show large impacts to the microbial community in regards to lipid composition in diet [82], may demonstrate that the honey bee microbiome is more resistant to changes in the complexity of the protein and hemicellulose components of pollen than it is to changes in the macromolecular composition of food items, including amino acids and lipids. Certainly, pollen contains many additional constituents lacking in our artificial formulation. Some of these include bioactive compounds like polyphenols and flavonoids that may modulate bacterial diversity through antimicrobial and antioxidant properties [85–87]. Thus, simulating the complex nature of pollen with nonfloral components is an elusive goal.

In conclusion, our most striking findings were the large impact of a pollen substitute on developmental gene expression and on resistance to infection. Our overall findings demonstrate that nutritional strategies for supplementing hives that include some proportion of pollen have more normal and robust microbiomes and more normal gene expression and immunity profiles. Further studies may reveal other host-related consequences of diets lacking natural pollen, such as the timing of a worker’s transition from performing in-hive tasks to foraging, and impacts to the expression of genes that are essential for maintaining colony health.

## Supporting information

S1 Fig Average per-capita consumption of food per diet type over time at TAMU. POL consumption slowed at a significantly greater rate than for the experimental diets (***p< 0.0001, ANOVA Repeated Measures Mixed Model Analysis). Data in S3 Table.
(TIF)

S2 Fig Cumulative consumption by diet group at TAMU. Bees in the POL group consumed less than bees in the other two groups and significantly less than those in the AD group. *p < 0.05, post hoc pairwise comparisons using Tukey and Kramer (Nemenyi) test with Tukey distribution approximation for independent samples following Kruskal-Wallis. Data in S3 Table.
(TIF)

S3 Fig Mortality over time for bees at TAMU by diet type. All groups had higher than 93% survival and none had significantly better or worse survivorship based on a Cox Proportional Hazards test. Data in S4 Table.
(TIF)

S4 Fig Effects of pollen (POL) versus artificial diet (AD) or artificial diet with additives (ADA) on absolute abundance of non-typical mostly environmental bacteria (ASVs binned as “other”). From bee guts at TAMU (A) and UT (B). *p < 0.05, **p < 0.01, ***p < 0.001, post hoc pairwise comparisons using Tukey and Kramer (Nemenyi) test with Tukey distribution approximation for independent samples following Kruskal-Wallis. Data in S1 Table.
(TIF)

S5 Fig Effects of pollen (POL) versus artificial diet (AD) or artificial diet with additives (ADA) on absolute abundance of bacterial lineages from bee guts at TAMU and UT. (A-B) *Bombella apis*, (C-D) *Bombilactobacillus* spp., (E-F) *Lactobacillus melliventris*, (G-H) *Bifidobacterium* spp., (I-J) *Snodgrassella alvi*. *p* < 0.05, **p < 0.01, ***p < 0.001, post hoc pairwise comparisons using Tukey and Kramer (Nemenyi) test with Tukey distribution approximation for independent samples following Kruskal-Wallis. Data in S1 Table.
(TIF)

S6 Fig Effects of pollen (POL) versus artificial diet (AD) or artificial diet with additives (ADA) on *Gillliamella* populations in bee guts at TAMU and UT. Absolute abundance of all associated *Gilliamella* ASVs based on 16S rRNA gene amplicons, from bee guts sampled at TAMU (A) and UT (B). Alpha diversity of *Gilliamella* strains as measured as effective species number (ESN) based on single gene (*rimM*) copy amplicons at TAMU (C) and UT (D). Absolute abundance as determined from *rimM* amplicon analysis of *G*. *apis* and *G*. *apicola* lineages at TAMU (E-F) and UT (G-H). *p* < 0.05, **p < 0.01, ***p < 0.001, post hoc pairwise comparisons using Tukey and Kramer (Nemenyi) test with Tukey distribution approximation for independent samples following Kruskal-Wallis. Data in S1 Table.
(TIF)

S7 Fig Effects of pollen (POL) versus artificial diet (AD) or artificial diet with additives (ADA) on alpha diversity of *Bifidobacterium* strains. ESN was assessed using ASVs of the single copy gene *groEL* sampled from bee guts at the TAMU site (A) and the UT site (B). No significant differences were observed between diet groups. Data in S1 Table.
(TIF)

S8 Fig The effect of presence or absence of conventional gut microbiota on gene expression of the insulin receptor genes *inR1* and *inR2* (relative to reference gene RPS5a) in heads and abdomens of 14-day-old workers. Bees were raised on pollen (n = 7 samples per condition per tissue). DC = bees fed a defined community of cultured isolates, MD = microbiota deficient bees that were allowed to emerge overnight from brood cells but not inoculated with gut microbiota. Relative expression of *inR1* in (A) bee heads and (B) abdomens. Relative expression of *inR2* in (C) bee heads and (D) abdomens. (Mann-Whitney Wilcoxon. *p < 0.05, **p < 0.01, ***p < 0.001). Data in S5 Table.
(TIF)

S9 Fig The impact of pollen (POL) versus artificial diet (AD) or artificial diet with additives (ADA) on insulin receptor gene expression relative to the reference gene RPS5a in abdomens from bees at TAMU and UT. (A-B) Relative mRNA expression of the gene *inR1*. (C-D) Relative mRNA expression of *inR2*. n = 7 bees from 2 cups per condition per site. Differences between control and treatment groups were tested via post hoc pairwise comparisons using Tukey and Kramer (Nemenyi) test with Tukey distribution approximation for independent samples following Kruskal-Wallis. Data in S1 Table.
(TIF)

S1 File Supplementary materials and methods. (DOCX)

S2 File Summary of statistical test results. (TXT)

S1 Table Two-site diet experiment data. This table includes metadata pertaining to samples, absolute abundance data for bacterial lineages, alpha diversity metrics, single copy gene data and gene expression data. Data from this table is relevant to the following figures: Figs 2–4, S4–S7, S9 Figs).
(XLSX)

S2 Table List of primers and PCR protocols. (XLSX)

S3 Table TAMU consumption data. Data from this table is relevant to S1 and S2 Figs.
(XLSX)

S4 Table TAMU mortality data. Data from this table is relevant to S3 Fig.
(XLSX)

S5 Table Relative gene expression data from *in vivo* ± BGM experiment. Data from this table is relevant to Fig 6 and S8 Fig.
(XLSX)

S6 Table Relative gene expression data from percent pollen in diet experiment. Data from this table is relevant to Fig 5.
(XLSX)

S7 Table CFU of *Serratia marcescens* strain KZ11 (KAN+) recovered from infected bee guts. Data from this table is relevant to Fig 7.
(XLSX)
