# Supplementary figures and images for: The microbiome and gene expression of honey bee workers are affected by a diet containing pollen substitutes
Source: PLoS One. 2023 May 19;18(5):e0286070. doi: 10.1371/journal.pone.0286070 (PMC10198554; doi:10.1371/journal.pone.0286070)

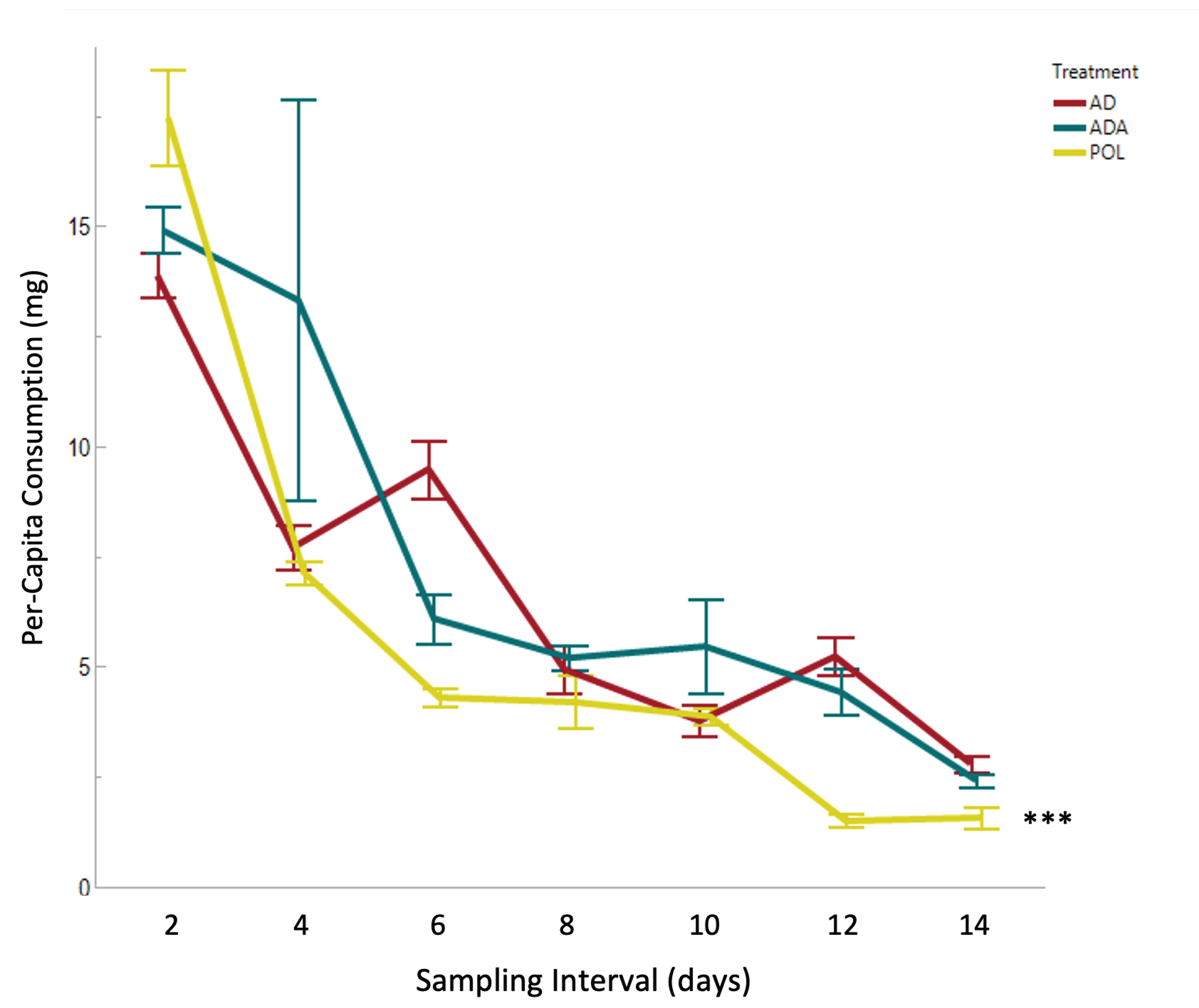

Supplement: S1 Fig — POL consumption slowed at a significantly greater rate than for the experimental diets (***p< 0.0001, ANOVA Repeated Measures Mixed Model Analysis). Data in S3 Table. (TIF) [file pone.0286070.s001.tif]

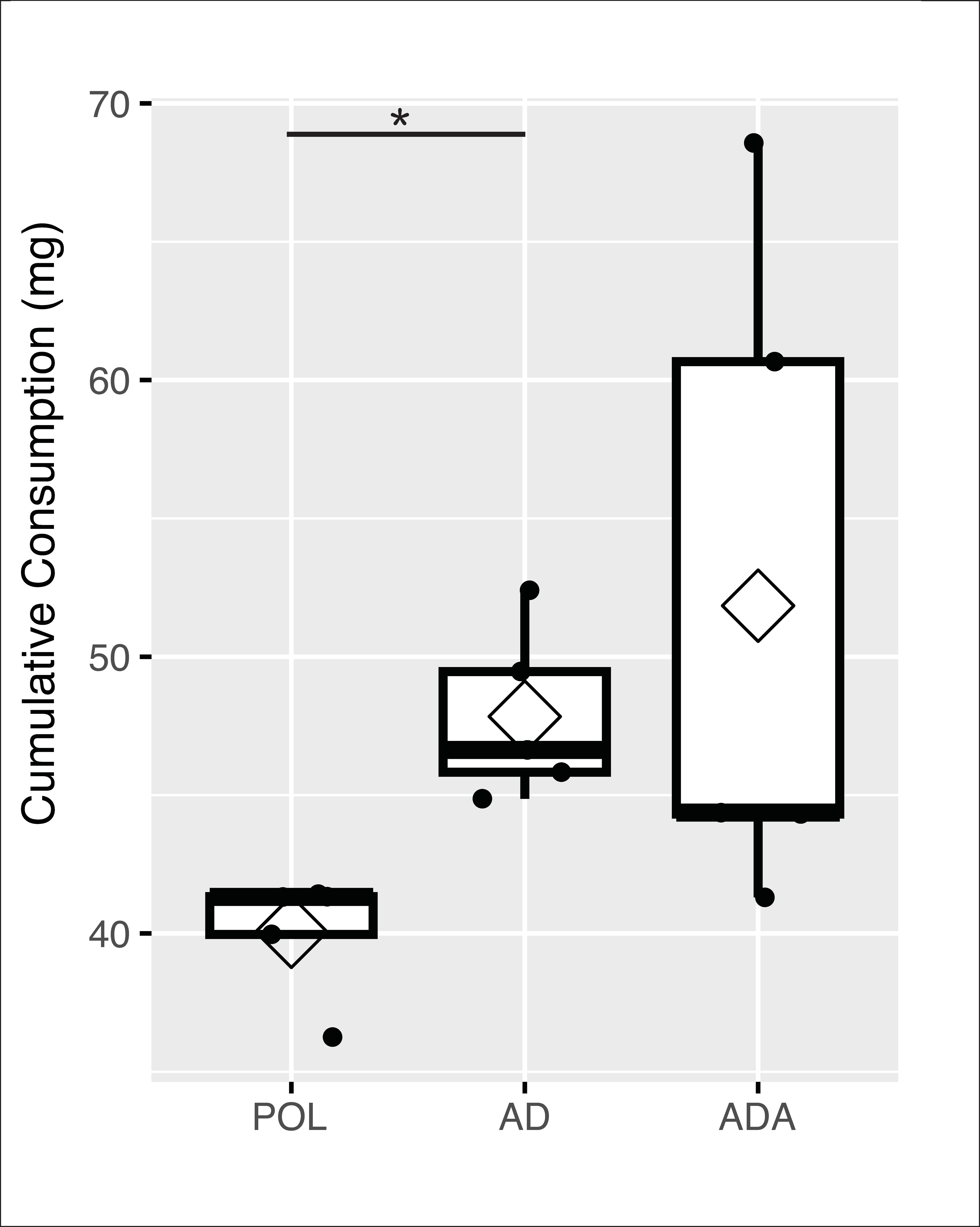

Supplement: S2 Fig — Bees in the POL group consumed less than bees in the other two groups and significantly less than those in the AD group. *p < 0.05, post hoc pairwise comparisons using Tukey and Kramer (Nemenyi) test with Tukey distribution approximation for independent samples following Kruskal-Wallis. Data in S3 Table. (TIF) [file pone.0286070.s002.tif]

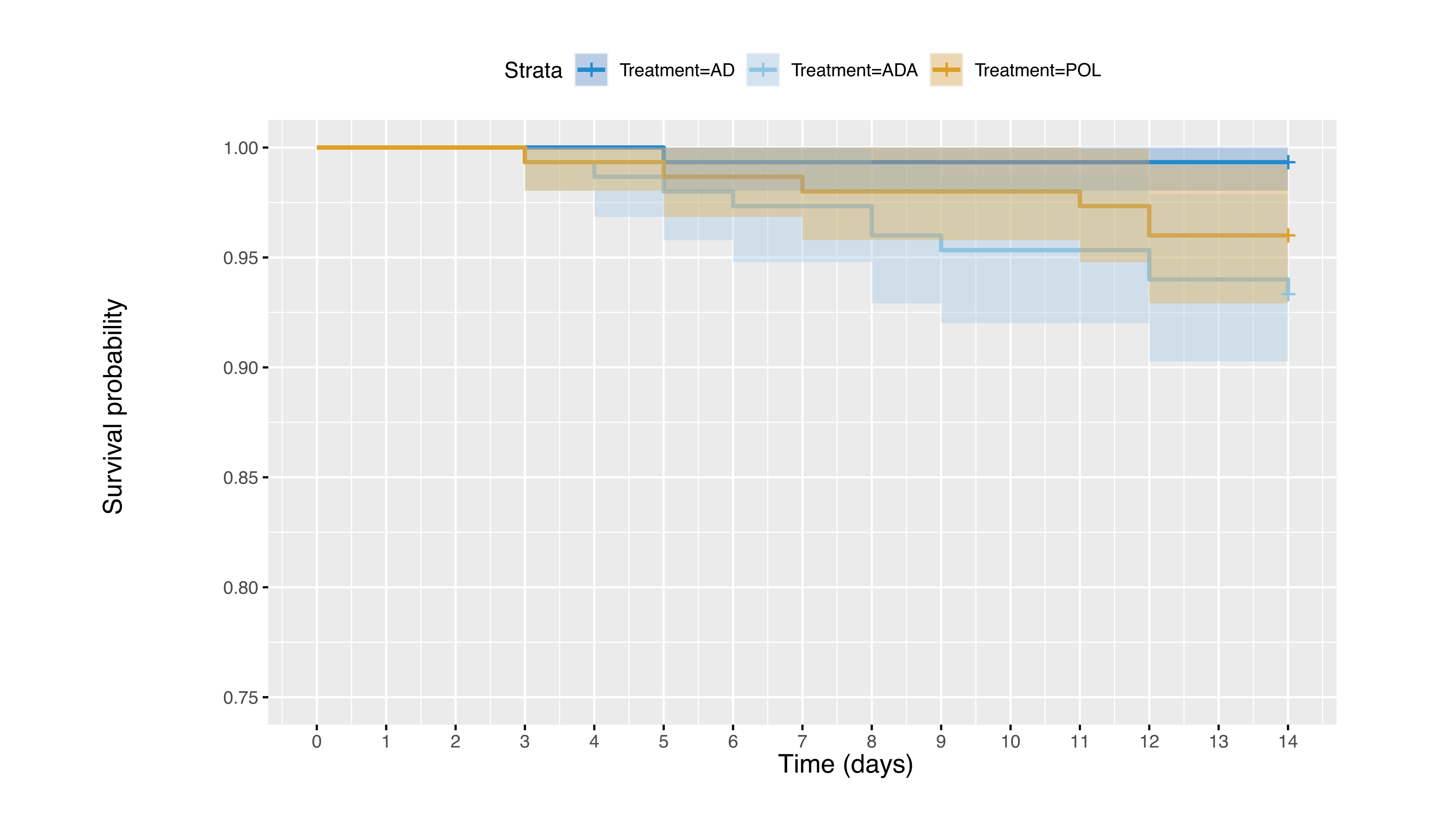

Supplement: S3 Fig — All groups had higher than 93% survival and none had significantly better or worse survivorship based on a Cox Proportional Hazards test. Data in S4 Table. (TIF) [file pone.0286070.s003.tif]

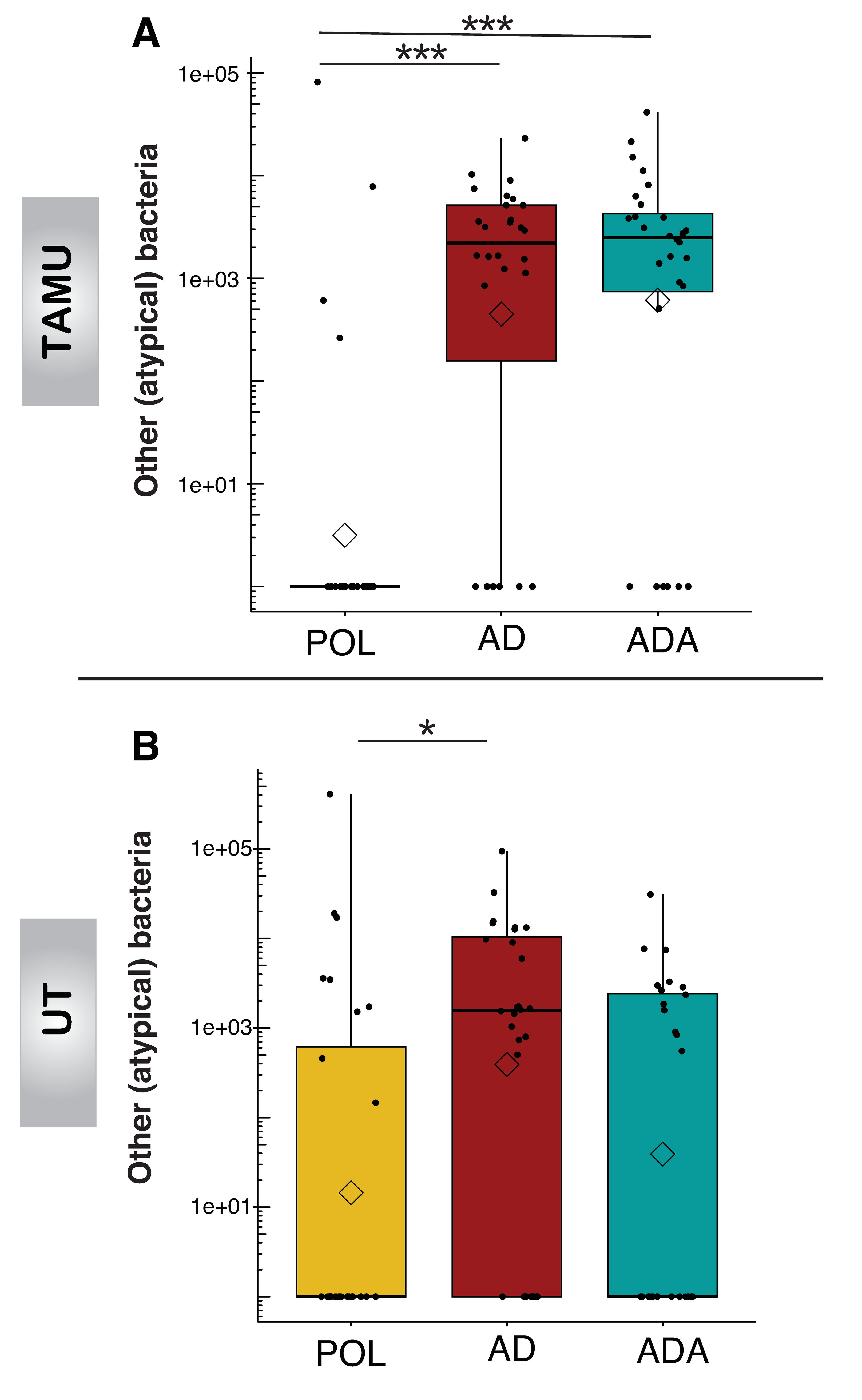

Supplement: S4 Fig — From bee guts at TAMU (A) and UT (B). *p < 0.05, **p < 0.01, ***p < 0.001, post hoc pairwise comparisons using Tukey and Kramer (Nemenyi) test with Tukey distribution approximation for independent samples following Kruskal-Wallis. Data in S1 Table. (TIF) [file pone.0286070.s004.tif]

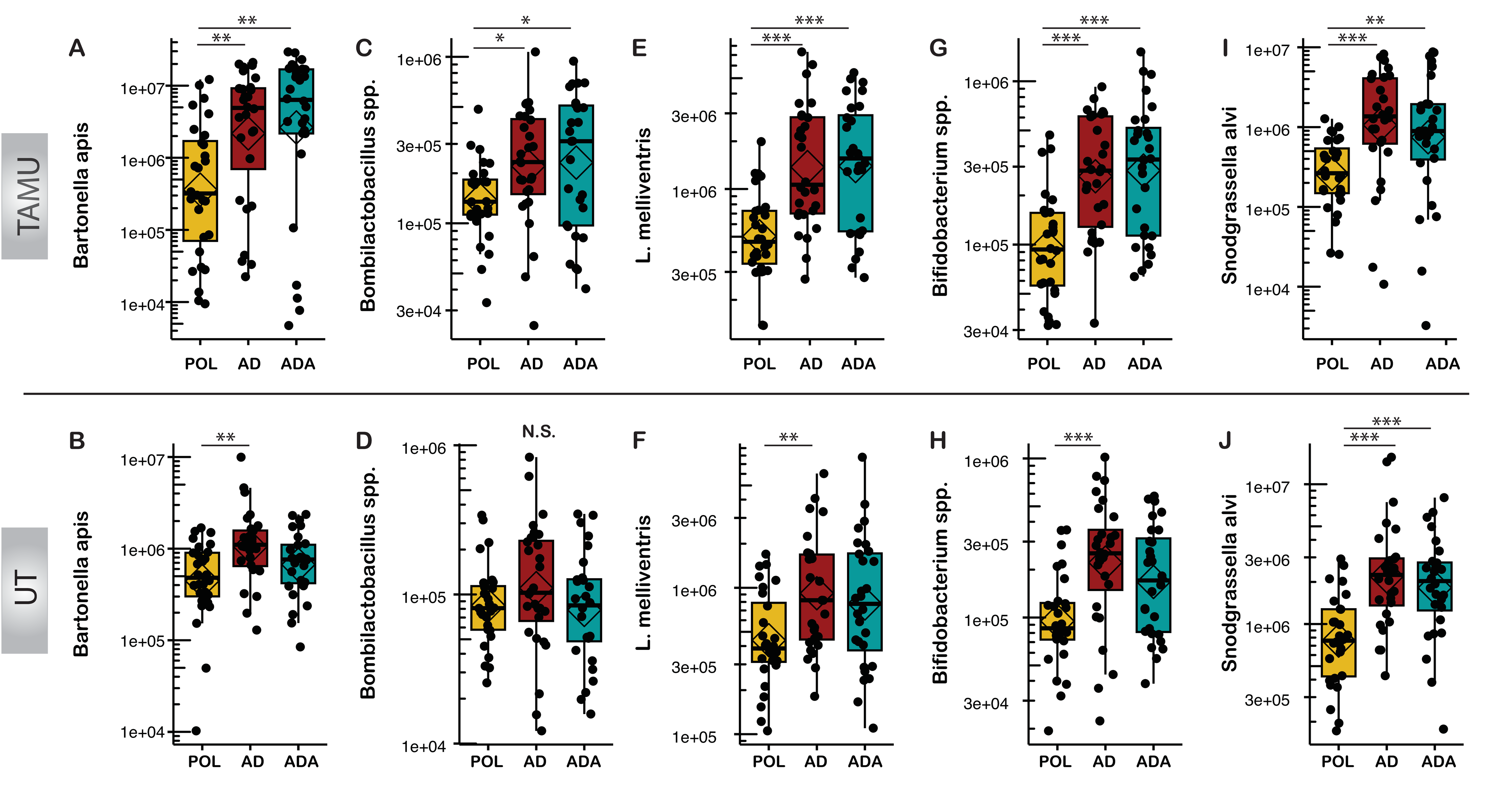

Supplement: S5 Fig — (A-B) Bombella apis, (C-D) Bombilactobacillus spp., (E-F) Lactobacillus melliventris, (G-H) Bifidobacterium spp., (I-J) Snodgrassella alvi. p < 0.05, **p < 0.01, ***p < 0.001, post hoc pairwise comparisons using Tukey and Kramer (Nemenyi) test with Tukey distribution approximation for independent samples following Kruskal-Wallis. Data in S1 Table. (TIF) [file pone.0286070.s005.tif]

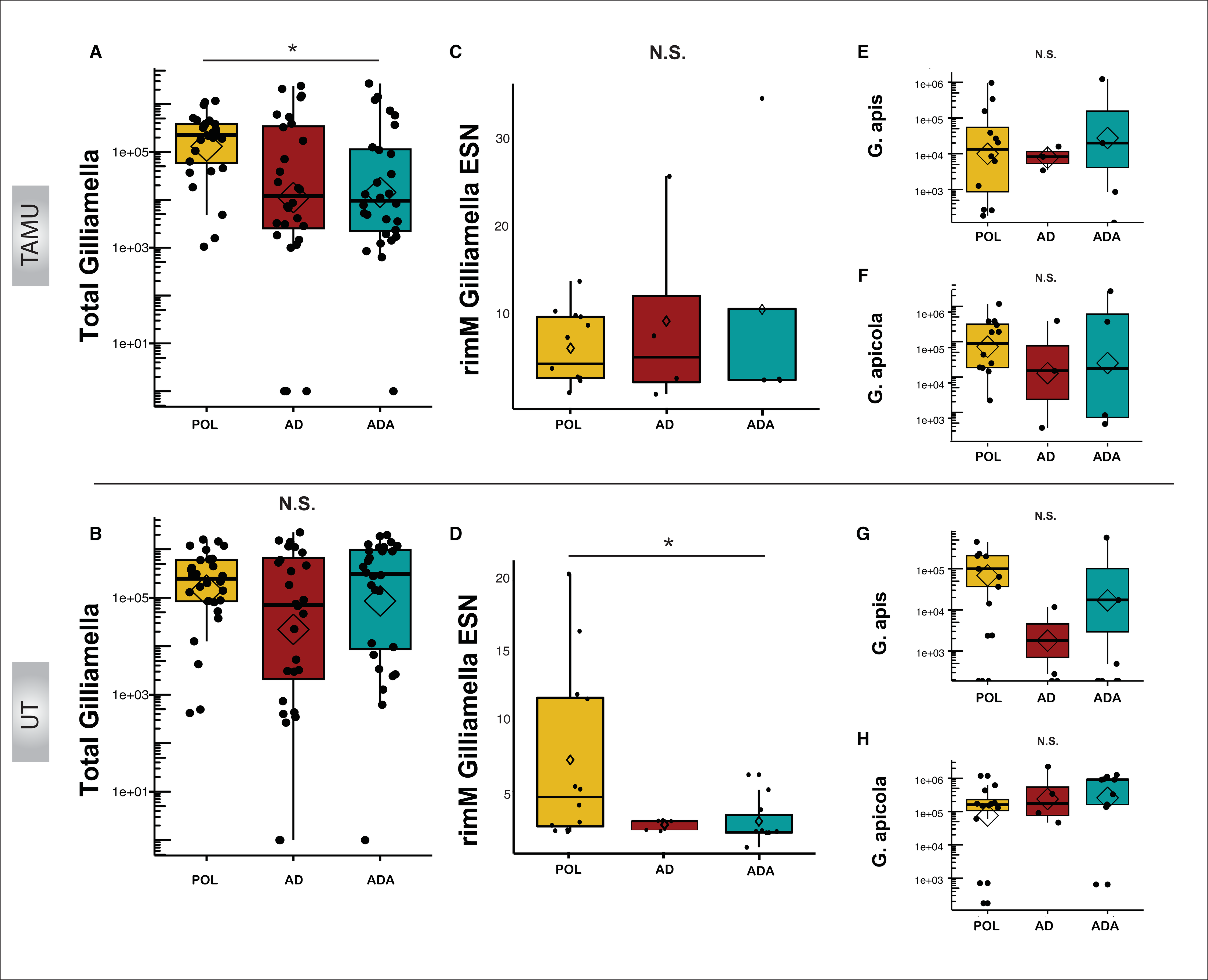

Supplement: S6 Fig — Absolute abundance of all associated Gilliamella ASVs based on 16S rRNA gene amplicons, from bee guts sampled at TAMU (A) and UT (B). Alpha diversity of Gilliamella strains as measured as effective species number (ESN) based on single gene (rimM) copy amplicons at TAMU (C) and UT (D). Absolute abundance as determined from rimM amplicon analysis of G. apis and G. apicola lineages at TAMU (E-F) and UT (G-H). p < 0.05, **p < 0.01, ***p < 0.001, post hoc pairwise comparisons using Tukey and Kramer (Nemenyi) test with Tukey distribution approximation for independent samples following Kruskal-Wallis. Data in S1 Table. (TIF) [file pone.0286070.s006.tif]

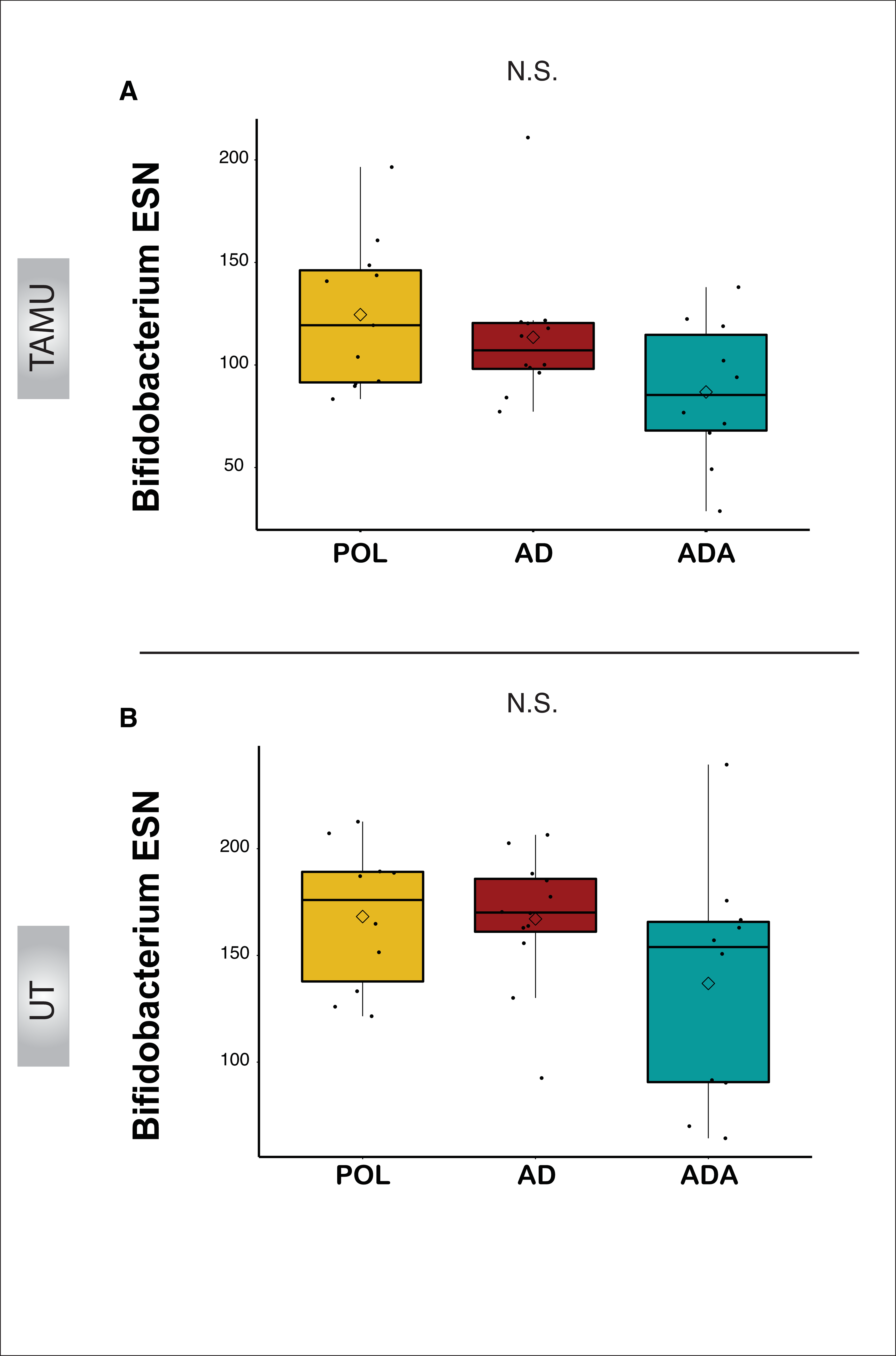

Supplement: S7 Fig — ESN was assessed using ASVs of the single copy gene groEL sampled from bee guts at the TAMU site (A) and the UT site (B). No significant differences were observed between diet groups. Data in S1 Table. (TIF) [file pone.0286070.s007.tif]

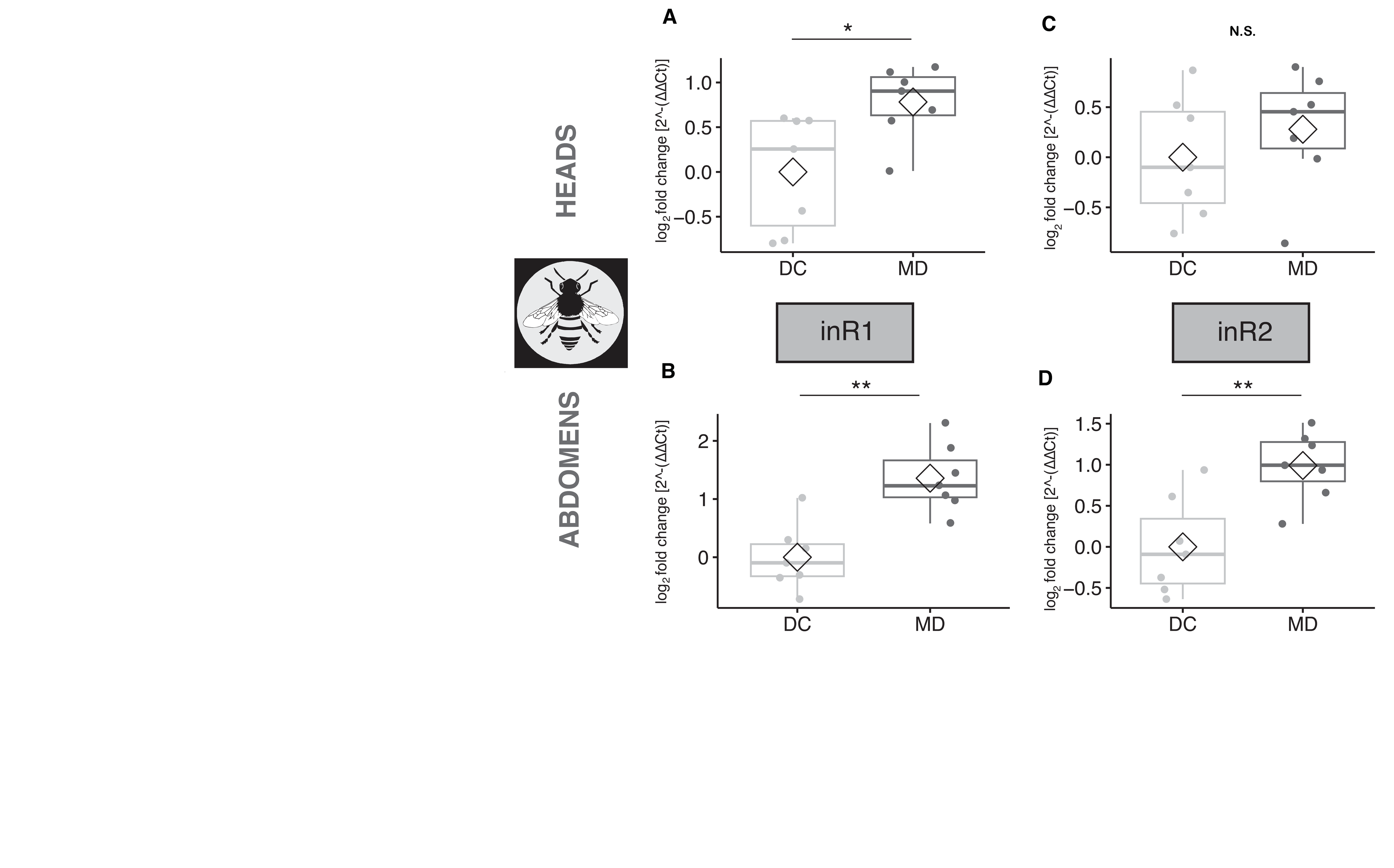

Supplement: S8 Fig — Bees were raised on pollen (n = 7 samples per condition per tissue). DC = bees fed a defined community of cultured isolates, MD = microbiota deficient bees that were allowed to emerge overnight from brood cells but not inoculated with gut microbiota. Relative expression of inR1 in (A) bee heads and (B) abdomens. Relative expression of inR2 in (C) bee heads and (D) abdomens. (Mann-Whitney Wilcoxon. *p < 0.05, **p < 0.01, ***p < 0.001). Data in S5 Table. (TIF) [file pone.0286070.s008.tif]

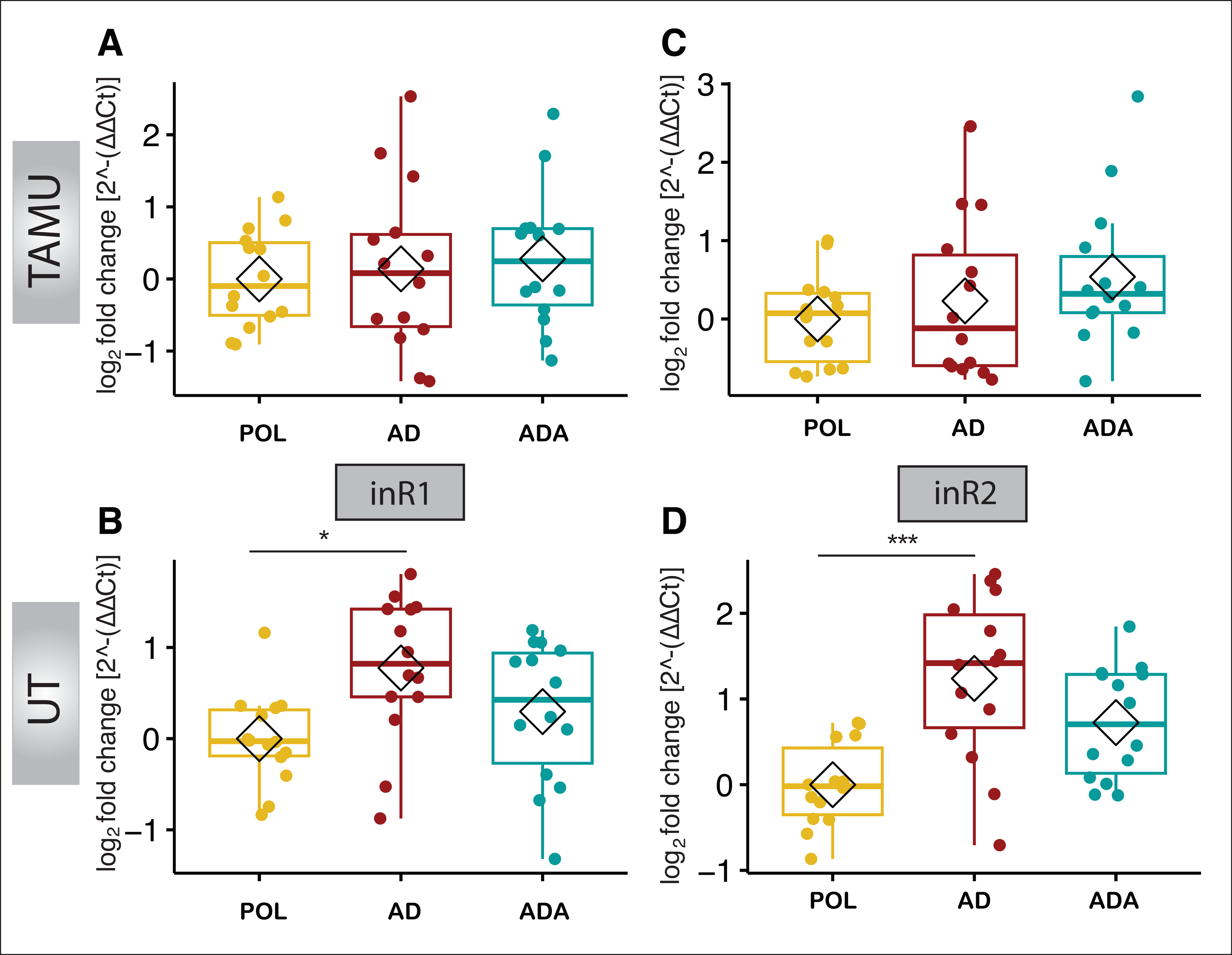

Supplement: S9 Fig — (A-B) Relative mRNA expression of the gene inR1. (C-D) Relative mRNA expression of inR2. n = 7 bees from 2 cups per condition per site. Differences between control and treatment groups were tested via post hoc pairwise comparisons using Tukey and Kramer (Nemenyi) test with Tukey distribution approximation for independent samples following Kruskal-Wallis. Data in S1 Table. (TIF) [file pone.0286070.s009.tif]
