# Supplementary material for: The microbiome and gene expression of honey bee workers are affected by a diet containing pollen substitutes
Source: PLoS One. 2023 May 19;18(5):e0286070. doi: 10.1371/journal.pone.0286070 (PMC10198554; doi:10.1371/journal.pone.0286070)
Supplement: S1 File — (DOCX) [file pone.0286070.s010.docx]

# **SUPPLEMENTARY METHODS**

## Artificial diet recipe

Ingredient list

AD

Isolated soy protein*

Linseed oil

Vanderzant vitamin mixture

Cellulose

Sucrose

ADA diet additions

Polygalacturonic acid

Beta glucan

Arabinan

Pectin galactan

Xyloglucan

*Calculations are based on the isolated soy protein powder from nuts.com, which contain 25g of protein and 1g of lipids per 28g of diet. The nutrient composition will vary depending on the brand and adjustments may need to be made.

- Add dry ingredients- isolated soy protein (33.6g), cellulose (10.5g), [*and for ADA only* polygalacturonic acid (10mg)]
- Create 50% w/v sucrose (41.4g) and mix in Vanderzant vitamin mixture (0.52g) [*and for ADA only* beta glucan (10mg), arabinan (10mg), pectin galactan (10mg), and xyloglucan (10mg)]
- Combine and mix wet ingredients, dry ingredients, and linseed oil (18.8g). A handheld mixer is recommended
- Store at -20°C and thaw before use

## High throughput sequencing of bacterial community

For community surveys of 16S V4 metabarcoded amplicons, the first PCR reaction amplified the diluted template in 20 μL reactions with Illumina-adapted 515F and 806R primers (See Table S2) [1] using Accustart 2 mastermix (Quanta Bio, MA, USA). Cycling conditions and mastermix recipes may be viewed in Table S2). These reactions were examined on a 2% agarose then purified with 0.8x HighPrep™ PCR magnetic beads (MAGBIO^®^, MD, USA). The cleaned product was diluted to a final volume of 52.5 μL. The second PCR reaction attached Illumina 8 bp Nextera style dual-indexed barcodes to 1 μL of PCR 1 product and was performed in 25 μL single reactions using a unique combination of N7XX and S5XX. These reactions were cleaned with magnetic beads, resuspended in 27.5 μL molecular grade water and quantified on a plate reader using the Accublue broad range dsDNA quantitation kit (Biotium, Fremont, CA, USA). Equimolar amounts of each amplification within each sampling interval were pooled, combined with 5% phiX DNA and sequenced on an Illumina iSeq instrument at 2 x 150 reads.

For taxon-specific single copy gene metabarcoding, we used two lineage-specific targets: for *Gilliamella* *rimM* [2], and for *Bifidobacterium* we used *groEL* [3]. We amplified each one of these gene targets in a single 25 ul reaction with Accustart 2 mastermix. Table S2 lists cycling conditions and master mix recipes. We screened, cleaned, barcoded and pooled the samples with the same strategy as with the 16S rRNA gene metabarcoding portion. We repeated any failed reactions with less dilute nucleic acid. We sent the pooled library to Admera Health by way of Genohub for sequencing via a miSeq 2x250 run (Illumina, CA, USA). Demultiplexed read data was returned to us for subsequent analysis.

Primer sequences and amplification protocols are listed in Table S2.

## Processing and analysis of high throughput reads

Demultiplexing of the iSeq 16S rRNA gene V4 metabarcode reads on the basis of barcode sequences was performed onboard the instrument by the iSeq software (Local Run Manager, Generate FASTQ Analysis Module 2.0). We then processed these split reads with QIIME 2 version 2020.2 [4]. Forward reads were used alone because not enough overlap existed between paired end sequences to assemble them. Primer and adapter sequences were removed with the *cutadapt* plugin [5]. Then they were truncated to 120 base pairs, filtered, denoised, and chimeric reads were removed using the *Deblur* plugin [6]. Taxonomy was assigned to amplicon sequence variants (ASVs) using the SILVA 132-99-515-806-nb database with the *feature-classifier* plugin [7]. We inspected ASVs and removed unassigned, mitochondrial, chloroplast, and singleton reads with the f*eature-table* plugin [8]. We used the *align-to-tree-mafft-fasttree* phylogenetic tree building function to reconstruct a phylogenetic tree to use with diversity estimation models [9]. For the visualization of composition within bee samples we combined ASVs within species groups and used a BLAST based binning strategy for the more complex lineages like *Lactobacillus* (made up of the bee specific clades of Firm-4 and Firm-5 as well as environmental Firmicutes).

For the 16S rRNA gene metabarcoded reads, alpha diversity was first examined by performing 10 subsampling iterations at every 100 reads at each sampling interval. We used a uniform depth of 3800 reads per sample was decided upon, as this depth preserved a high number of samples for examination and showed saturation of alpha diversity. We looked at two main measures of alpha diversity in an effort to decipher the scope of changes that may have occurred with treatment and subsequent recovery. We examined species richness by obtaining the Shannon index for each sample and then using this to calculate the Effective Species Number (ESN) [10]. We further explored evenness within communities per sample by using Pielou’s evenness index [11]. We compared differences between groups by using Kruskal-Wallis rank sum tests and post-hoc multiple comparisons tests in R with the kruskal.mc command from the *pgirmess* package [12] and Tukey and Kramer (Nemenyi) test with Tukey distribution approximation for independent samples from the R package PMCMR [13]. We plotted charts in R using *ggplot2*[14]. We compared beta diversity between groups by using weighted UniFrac [15] distance matrices and testing for significance with PERMANOVA tests.

We used the relative abundances found by analyzing groups of taxa in amplicon sequence variant (ASV) tables to calculate the absolute abundance of bacterial lineages. We divided the total number of 16S rRNA gene copies estimated from qPCR by the relative abundance proportions for each taxa and corrected for rRNA operon number per genome as done in [16]. These data are included in S1 Table. For statistical analysis and representation purposes, we added the number one to all entries in the absolute abundance table. We did this as many lineages had entries for samples with zero absolute abundance and these were not mathematically defined in logarithmic analysis. We then compared the absolute abundance of each lineage with Kruskal-Wallis rank sum tests and post hoc tests as mentioned above.

For the taxon-specific single-copy gene method, we used a similar set of techniques as with the 16S rRNA gene V4 metabarcoding portion. Only samples that produced positive amplifications were sent for sequencing. In an alteration to the preceding strategy, we used longer paired-end reads (2x250) and so were able to assemble the *rimM* sequences into longer high-quality sequences with the *vsearch/join-pairs* plugin for Qiime2.The reverse reads for the groEL sequences were low quality and so we used single forward reads to conduct the analysis. These assembled sequences were then trimmed to the longest high-quality length that yielded the largest pool of total reads for each gene target. We also chose trim lengths which were within the sequenced portion of enough GenBank deposited sequences to allow for some confidence in phylogenetic tree inference and strain-level taxonomic assignment. The trim length for *rimM* (*Gilliamella*) 295 bp and for *groEL* (*Bifidobacterium*) 145 bp. To perform these strain-level taxonomic analyses, we had to first construct databases (used in [17]) and train classifiers for each gene target of deposited variants that were within a range of similarity to one another ~99.9 – 83%. These variants were retrieved, trimmed to the region of comparison, aligned, and then used to train classifiers with the *feature-classifier/fit-classifier-naive-bayes* plugin in Qiime2. For the rimM reads we had to filter out contamination from *Frischella perrara* (a close relative of *Gilliamella* spp.) from the sample reads. Alpha and Beta diversity analyses were then performed in the same manner as with the 16S rRNA gene V4 portion though subsampling levels were calculated per gene target based on read depth and saturation (*rimM* = 500 reads, *groEL*_Bifido = 100 reads).

For both of these targets we focused on evaluating the alpha diversity measure of ESN at each site and treatment. For *rimM* analyses we performed one ESN analysis at the genus level and then further split reads to the species level (*G. apis* and *G. apicola*) by inferring a phylogenetic tree with sequences from our initial alignments and ASVs of representative sequences utilizing the default workflow from (http://www.phylogeny.fr/advanced.cgi). We then performed examined ESN at this species level at each site. The short reads and low read depth used in the *Bifidobacterium* groEL analysis did not allow for this species-level analysis and so we ESN at the genus level.

In order to examine differential abundances in taxonomic groups that may be driving differences between treatment groups diversity we employed the ANCOM compositional analysis tool [18] in Qiime2. In order to use ANCOM we first collapsed each timepoint’s taxonomic tables at level 5 and added a pseudo count before applying the tool. Differences flagged by ANCOM were explored by statistically comparing absolute abundance for each lineage between treatment groups at each sampling point using the nonparametric approach mentioned above.

## qPCR for 16S rRNA gene copy abundance

We used the techniques outlined in previous studies [19,20] for absolute SYBR green qPCR quantitation of total 16S rRNA gene copies with the 27F/355R (See Table SI2) [21] Universal 16S primer set along with a serially diluted plasmid-based standard.

Resultant 16S rRNA gene copy counts were corrected for dilution and then the estimated absolute abundance for each bacterial species was calculated in the method mentioned previously.

## REFERENCES

1. Wang Y, Qian PY. Conservative fragments in bacterial 16S rRNA genes and primer design for 16S ribosomal DNA amplicons in metagenomic studies. PLoS One. 2009;4(10).

2. Raymann K, Bobay L-M, Moran NA. Antibiotics reduce genetic diversity of core species in the honeybee gut microbiome. Mol Ecol. 2017;26:2057–66.

3. Hu L, Lu W, Wang L, Pan M, Zhang H, Zhao J, et al. Assessment of Bifidobacterium species using groEL gene on the basis of illumina miseq high-throughput sequencing. Genes. 2017;8(11).

4. Bolyen E, Rideout JR, Dillon MR, Bokulich NA, Abnet CC, Al-Ghalith GA, et al. Reproducible, interactive, scalable and extensible microbiome data science using QIIME 2. Nat Biotechnol. 2019;37(8):852–7.

5. Martin M. Cutadapt Removes Adapter Sequences From High-Throughput Sequencing Reads. EMBnet.journal. 2011;17(1):10–2.

6. Amir A, Daniel M, Navas-Molina J, Kopylova E, Morton J, Xu ZZ, et al. Deblur Rapidly Resolves Single Nucleotide Community Sequence Patterns. Abstr Gen Meet Am Soc Microbiol. 2017;2(2):1–7.

7. Bokulich NA, Kaehler BD, Rideout JR, Dillon M, Bolyen E, Knight R, et al. Optimizing taxonomic classification of marker-gene amplicon sequences with QIIME 2’s q2-feature-classifier plugin. Microbiome. 2018;6(1):1–17.

8. Estaki M, Jiang L, Bokulich NA, McDonald D, González A, Kosciolek T, et al. QIIME 2 Enables Comprehensive End-to-End Analysis of Diverse Microbiome Data and Comparative Studies with Publicly Available Data. Curr Protoc Bioinformatics. 2020;70(1):1–46.

9. Katoh K, Misawa K, Kuma KI, Miyata T. MAFFT: A novel method for rapid multiple sequence alignment based on fast Fourier transform. Nucleic Acids Res. 2002;30(14):3059–66.

10. Jost L. Entropy AndDiversity. Oikos. 2006;2(113).

11. Pielou EC. The measurement of diversity in different types of biological collections Vol. 15, J Theor Biol. 1967. p. 177.

12. Giraudoux P. R software package ‘ pgirmess ’. [software]. 2017. Available from: https://cran.r-project.org/web/packages/pgirmess/index.html

13. Pohlert T. The Pairwise Multiple Comparison of Mean Ranks Package (PMCMR). [software].2014. Available from: https://CRAN.R-project.org/package=PMCMR

14. Wickham H. ggplot2 Elegant Graphics for Data Analysis. New York, USA: springer-Verlag; 2009.

15. Lozupone C, Lladser ME, Knights D, Stombaugh J, Knight R. UniFrac: An effective distance metric for microbial community comparison. ISME J. 2011;5(2):169–72.

16. Raymann K, Shaffer Z, Moran NA. Antibiotic exposure perturbs the gut microbiota and elevates mortality in honeybees. PLoS Biol. 2017;15(3):e2001861.

17. Powell JE, Carver Z, Leonard SP, Moran NA. Field-Realistic Tylosin Exposure Impacts Honey Bee Microbiota and Pathogen Susceptibility, Which Is Ameliorated by Native Gut Probiotics. Microbiol Spectr. 2021;9(1).

18. Mandal S, Van Treuren W, White RA, Eggesbø M, Knight R, Peddada SD. Analysis of composition of microbiomes: a novel method for studying microbial composition. Microb Ecol Health Dis. 2015;26(0):1–7.

19. Martinson VG, Moy J, Moran NA. Establishment of characteristic gut bacteria during development of the honeybee worker. Appl Environ Microbiol. 2012;78(8):2830–40.

20. Powell JE, Martinson VG, Urban-Mead K, Moran NA. Routes of acquisition of the gut microbiota of the honey bee Apis mellifera. Appl Environ Microbiol. 2014;80(23):7378–87.

21. Castillo M, Martín-Orúe SM, Manzanilla EG, Badiola I, Martín M, Gasa J. Quantification of total bacteria, enterobacteria and lactobacilli populations in pig digesta by real-time PCR. Vet Microbiol. 2006;114(1–2):165–70.
